# Functional Role of Mst1/Mst2 in Embryonic Stem Cell Differentiation

**DOI:** 10.1371/journal.pone.0079867

**Published:** 2013-11-05

**Authors:** Peng Li, Ying Chen, Kinglun Kingston Mak, Chun Kwok Wong, Chi Chiu Wang, Ping Yuan

**Affiliations:** 1 Department of Chemical Pathology, the Chinese University of Hong Kong, Prince of Wales Hospital, Shatin, New Territories, Hong Kong SAR, China; 2 Li Ka Shing Institute of Health Sciences, the Chinese University of Hong Kong, Prince of Wales Hospital, Shatin, New Territories, Hong Kong SAR, China; 3 School of Biomedical Sciences, the Chinese University of Hong Kong, Shatin, New Territories, Hong Kong SAR, China; 4 CUHK Shenzhen Research Institute, the Chinese University of Hong Kong, Shenzhen, Guangdong, China; 5 Key Laboratories for Regenerative Medicine, Ministry of Education, the Chinese University of Hong Kong, Shatin, New Territories, Hong Kong SAR, China; 6 Fetal Medicine Unit, Department of Obstetrics and Gynaecology, the Chinese University of Hong Kong, Prince of Wales Hospital, Shatin, New Territories, Hong Kong SAR, China; Northwestern University, United States of America

## Abstract

The Hippo pathway is an evolutionary conserved pathway that involves cell proliferation, differentiation, apoptosis and organ size regulation. Mst1 and Mst2 are central components of this pathway that are essential for embryonic development, though their role in controlling embryonic stem cells (ES cells) has yet to be exploited. To further understand the Mst1/Mst2 function in ES cell pluripotency and differentiation, we derived *Mst1/Mst2* double knockout (*Mst-/-*) ES cells to completely perturb Hippo signaling. We found that *Mst-/-* ES cells express higher level of Nanog than wild type ES cells and show differentiation resistance after LIF withdrawal. They also proliferate faster than wild type ES cells. Although *Mst-/-* ES cells can form embryoid bodies (EBs), their differentiation into tissues of three germ layers is distorted. Intriguingly, *Mst-/-* ES cells are unable to form teratoma. *Mst-/-* ES cells can differentiate into mesoderm lineage, but further differentiation to cardiac lineage cells is significantly affected. Microarray analysis revealed that ligands of non-canonical Wnt signaling, which is critical for cardiac progenitor specification, are significantly repressed in *Mst-/-* EBs. Taken together our results showed that Mst1/Mst2 are required for proper cardiac lineage cell development and teratoma formation.

## Introduction

The Hippo pathway was first discovered in *Drosophila*. Through genetic mosaic screens, core components of the Hippo pathway, such as *Warts* (*Wts*), *Hippo* (*Hpo*) and *Salvador* (*Sav*) were identified as tumor-suppressor genes [1-4]. These components restrict cell proliferation and promote apoptosis by repressing the downstream effector *Yokie* (*Yki*) *i*n Drosophila. Depletion of core components of the Hippo pathway or overexpression of *Yki* results in enhanced cell proliferation and reduced apoptosis respectively [5]. This pathway is highly conserved in mammals. Serine/threonine kinases Mst1/Mst2 and Lats1/Lats2 in mammals are homologs of Hippo and Wts in *Drosophila* respectively. Together with an adaptor protein hMob1, they transmit signals to downstream effectors [6]. Through inhibiting the transcriptional co-activators and oncoproteins Yap (Yes kinase-associated protein) and Taz (transcriptional coactivator with PDZ-binding motif), the Hippo pathway promotes apoptosis and inhibits tumorigenesis in mammals [7-10].

Mst1 and Mst2 (Mammalian sterile 20-like kinases 1 and 2) are the core components of the Hippo pathway. They play important roles in early embryonic development, cell proliferation, apoptosis and organ size control. *Mst1* null mice are viable and fertile but have a reduced number of mature naive T cells, while *Mst2* null mice are also fertile but exhibit no developmental or immunological defects [11]. However, depletion of both *Mst1* and *Mst2* resulted in embryonic lethality at embryonic day 8.5, suggesting redundant roles of *Mst1* and *Mst2* [12]. One functional copy of either *Mst1* or *Mst2* is necessary and sufficient for early embryonic development [11,13].

Like other components of the Hippo pathway that promote apoptosis, Mst1/Mst2 are pro-apoptotic kinases [14,15]. Under oxidative stress, Mst1/Mst2 activate transcription factor Foxo and promote neuronal cell death [16,17]. Heart specific expression of *Mst1* leads to dilated cardiomyopathy with reduction in cell density in heart [18]. Liver specific removal of *Mst1/Mst2* in newborn mice results in liver enlargement and formation of hepatocellular carcinoma and cholangiocarcinoma [12,19-21]. Similarly, in mouse intestines and pancreas, inactivation of *Mst1/Mst2* leads to intestinal stem cell overproliferation, colonic tumorigenesis and pancreas overgrowth [22-24], suggesting important roles of Mst1/Mst2 in organ size control and tumorigenesis. 

Mst1/Mst2 activate Lats1 and Lats2 by phosphorylation, and in turn phosphorylate Yap and inhibit it from translocating into the nucleus [25]. Unphosphorylated Yap can be translocated into the nucleus to activate TEA-domain (TEAD) family members. The Yap/Taz-Tead complex further activates proliferation by a genome wide transcriptional program [26-28]. Ectopic expression of *Yap* in mammalian cells leads to a phenotype resembling that from ablation of core components of the Hippo pathway. Similar to the simultaneous removal of *Mst1/Mst2*, overexpression of *Yap* in mice results in a dramatic increase of liver mass with subsequent tumor formation. In addition previous research reveals that Yap is an important pluripotent factor. Expression of *Yap* enhances reprogramming of differentiated cells to induced pluripotent stem (iPS) cells [26,29,30]. In adults Yap is enriched in organs such as the small intestine and the developing brain and its expression is highly restricted to the progenitor or stem compartments, whereas in other tissues, such as skin and skeletal muscle, the expression of *Yap* is gradually decreased with regard to differentiation status [10,26,31,32]. *Yap* is therefore, a stemness gene in mammalian cells, while key components of Hippo pathway such as Mst1/Mst2, function to constrain this stemness gene in restricted compartments. 

*Mst1/Mst2* double knockout mice die at E8.5 with abnormalities in the placenta, vascular patterning and primitive hematopoiesis, suggesting that Mst1/Mst2 are not required for pluripotent inner cell mass (ICM) formation but are required for subsequent organ and tissue development. As an *in vitro* derivative of the pluripotent inner cell mass (ICM), ES cells retain the developmental characteristics of ICM and can self-renew and differentiate to all three germ layers. When ES cells are injected to the blastocysts, they can contribute to all of the animal cell types [33,34]. These unique properties make ES cells suitable for genetic modification and they have the potential to serve as a source of regenerative medicine for cell therapy. In addition, successful reprogramming of somatic cells into induced pluripotent stem cells opens a new gate for stem cell therapy that avoids various ethical issues [35-37]. Direct transplanting of ES cells into hosts can however lead to teratoma formation and this remains a clinical challenge for ES cell application. Harnessing this valuable tool for therapeutic use will require overcoming this problem through innovative exploitation of the mechanisms involved in ES cell pluripotency, differentiation and tumorigenesis. Many signaling pathways, including the Hippo pathway in ES cell pluripotency and lineage commitment, have yet to be well characterized. Mst1/Mst2 are however required for proper development of some organs and tissue in embryos, but it is not clear whether Mst1/Mst2 play any corresponding role during ES cell differentiation at cellular level that consequently leads to developmental defects. To further address this question, we generated *Mst-/-* ES cells from *Mst1* and *Mst2* mutant mice [12]. We found that the phosphorylation level of Yap was decreased in *Mst-/-* ES cells, whilst the pluripotency marker Nanog was increased significantly compared to wild type ES cells. *Mst-/-* ES cells also showed differentiation resistance for a relatively longer time compared to wild type ES cells under differentiation conditions. Consistent with the developmental defects of *Mst1/Mst2* double knockout mice, *Mst-/-* ES cells showed lineage development distortion during embryoid body (EB) formation and obvious defects to differentiation to cardiac progenitor cells. Microarray analysis revealed that the non-canonical Wnt pathway ligands *Wnt2b* and *Wnt5a*, which are critical for cardiac progenitor cell differentiation, were significantly downregulated in *Mst-/-* EBs. Unlike wild type ES cells, *Mst-/-* ES cells could not form teratoma after subcutaneously injected into nude mice. Taken together, our data suggest that Mst1/Mst2 are required for teratoma formation. Their functions are also critical for proper cardiac lineage cell formation. 

## Results

### Derivation of mouse Mst-/- embryonic stem cells

**Figure 1:**
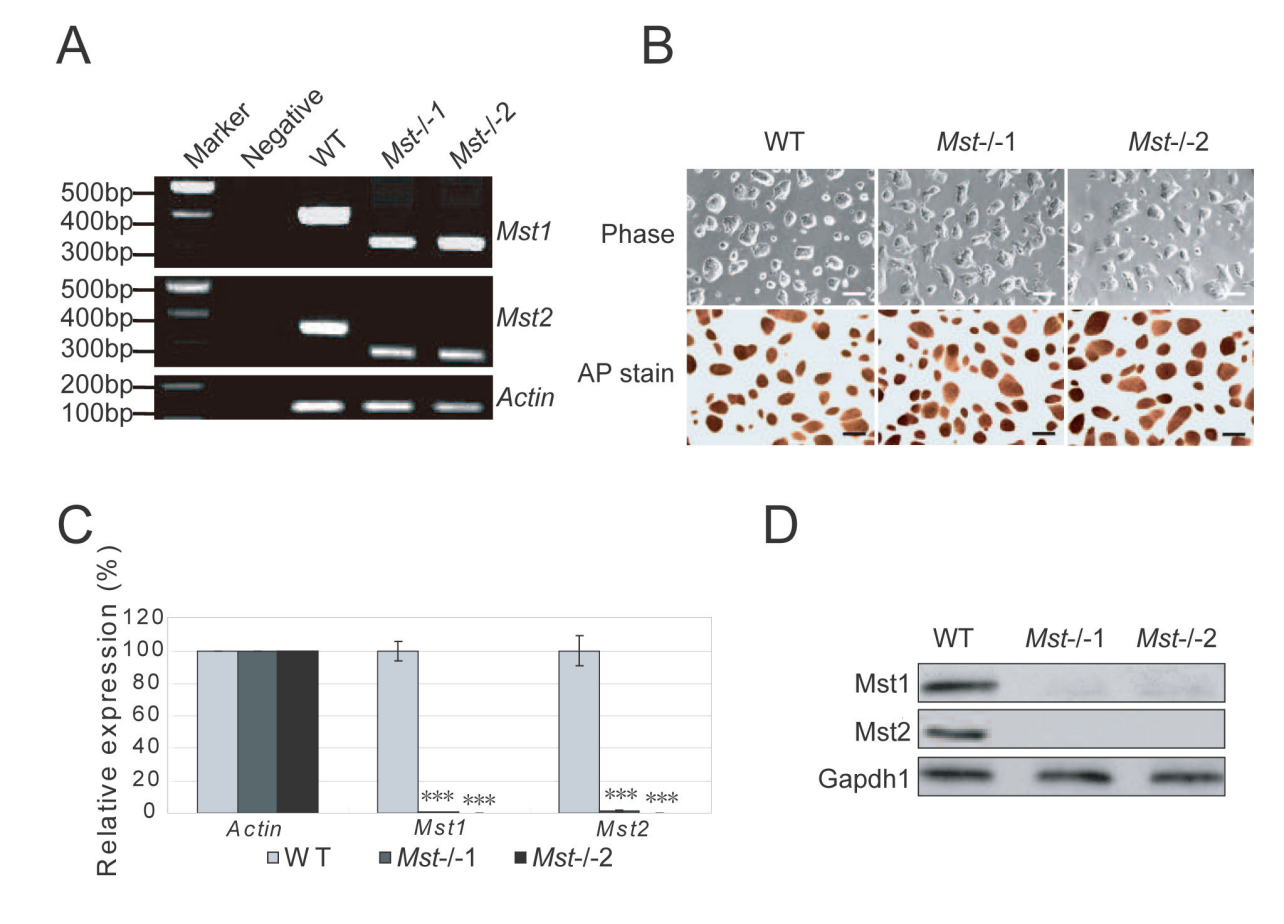
Isolation of *Mst-/-* ES cells. (A) Genotyping of wild type (WT) ES cells and *Mst-/*- ES cells derived from blastocysts by PCR amplification of genomic DNA. Wild type ES cells showed a larger band while *Mst-/*- ES cells displayed a smaller band. *Actin* was used as an internal control. (B) Phase contrast microscopy of wild type (WT) and two independent *Mst-/*- knockout ES cell lines (*Mst-/-*1 and *Mst-/-*2) grown on 0.2% gelatin in 2i+LIF medium (Upper). These cells were stained for alkaline phosphatase (Lower). Scale bar, 200 μm. (C) mRNA level of *Mst1* and *Mst2* in wild type ES cells and *Mst-/*- ES cells examined by quantitative real-time PCR using primers flanking the deleted region of *Mst*1 and *Mst*2. The data are shown as the mean ± S.D (n=3). *Actin* was normalized as an internal control. Statistically significant differences are indicated (*, P<0.05; **, P<0.01; ***, P<0.001). (D) Immunoblotting analysis of the expression of Mst1 and Mst2 in wild type ES cells and *Mst-/*- ES cells. Gapdh1 was used as a loading control.

Based on the schematics of null alleles of *Mst1* and *Mst2* generated in a previous study [12], we crossed the *Mst1* ^*+/-*^Mst2^-/-^ male and female mice, harvested the E3.5 embryos and derived ES cells on MEF feeder (Figure S1A and S1B). These cells were further adapted to form feeder-free ES cell lines under the 2i+LIF condition for genotyping. Exon 4 and 5 which encode kinase domain are deleted in Mst1 knockout, while exon 5 and 6 which encode kinase domain are deleted in Mst2 knockout (Figure S1C). With primers targeted to the adjacent sequence of the deleted regions of *Mst1* (exon 4 and 5) and *Mst2* (exon 5 and 6), PCR confirmed that respective regions of *Mst1* and *Mst2* genomic DNA were deleted in *Mst1*/*Mst2* double knockout (*Mst-/-*) ES cell lines respectively (Figure 1A). Two *Mst1*/*Mst2* double knockout lines, *Mst-/-*1 and *Mst-/-*2 ES cell lines were selected for further studies (Figure 1A). The *Mst-/-* ES cells still maintained dome-shaped colony morphology similar to that of the wild type ES cells (Figure 1B). They also expressed high levels of ES protein alkaline phosphatase (Figure 1B). To further confirm the absence of both *Mst1* and *Mst2* in the *Mst-/-* ES cells, primers targeting the corresponding deleted transcript region were used to do RT-PCR, the result confirmed that neither *Mst1* nor *Mst2* transcript was found in *Mst-/-* ES cells (Figure 1C). Further examination of the protein extracts with antibodies specifically against kinase domains of Mst1 and Mst2 protein showed that neither Mst1 nor Mst2 proteins were detected in the *Mst-/-* ES cells (Figure 1D). Taken together, these results showed that both *Mst1* and *Mst2* were functionally inactive in both *Mst-/-* ES cell lines.

### Characterization of mouse Mst-/- embryonic stem cells

**Figure 2:**
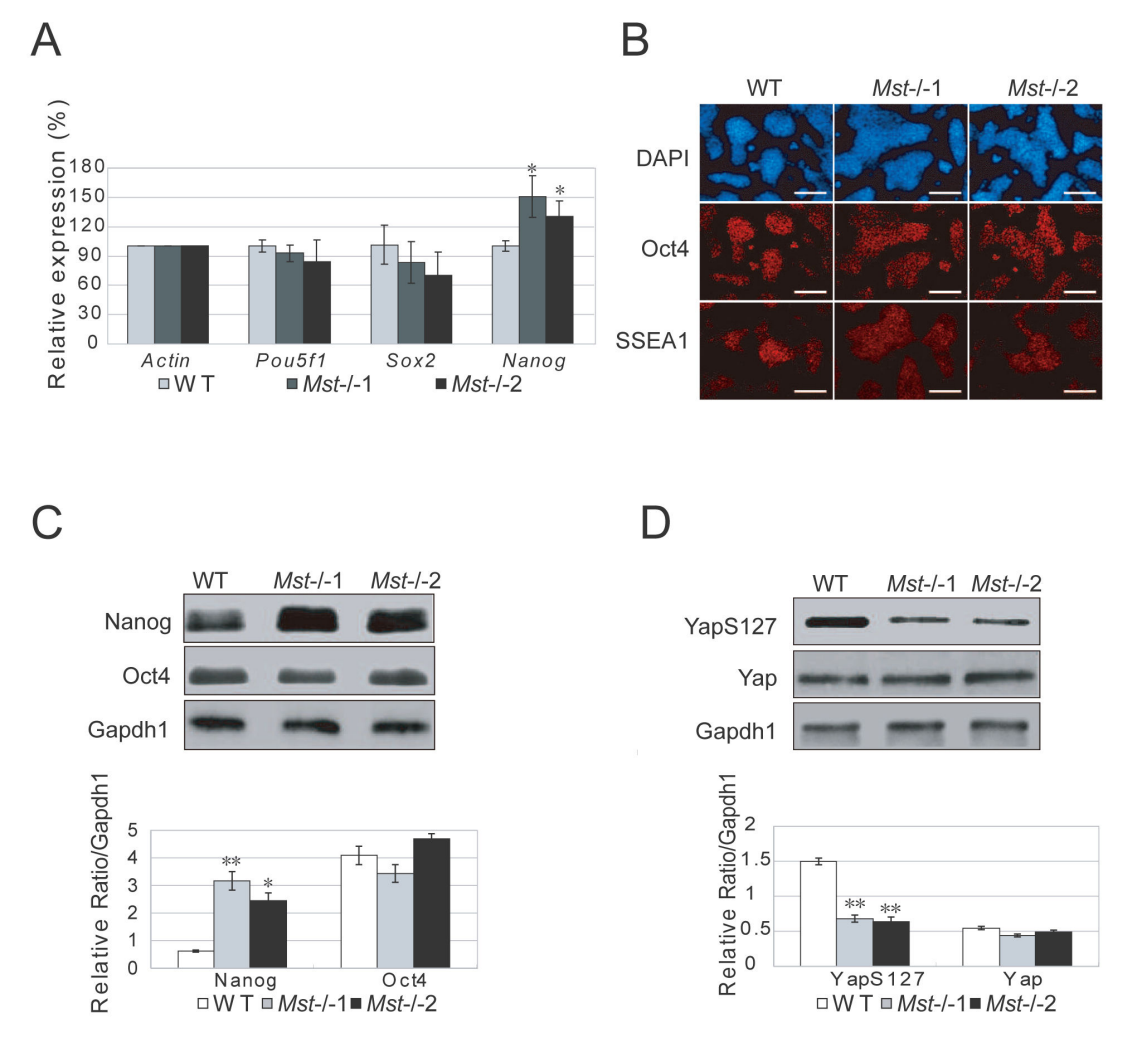
Characterization of Mst-/- ES cells. (A) Quantitative real-time PCR to examine the mRNA level of pluripotent markers *Pou5f1*, *Sox2* and *Nanog* in wild type ES cells and *Mst-/*- knockout ES cells. *Actin* was analyzed as an internal control. The data are shown as the mean ± S.D (n=3). Statistically significant differences are indicated (*, P<0.05; **, P<0.01; ***, P<0.001). (B) Immunofluorescence staining of the pluripotent protein Oct4 and SSEA1 expression in wild type ES cells and *Mst-/*- knockout ES cells. Neuclei were stained with DAPI. Scale bar, 200μm. (*C*) Immunoblotting and densitometric analysis of Nanog and Oct4 in wild type ES cells and *Mst-/*- ES cells. Gapdh1 was analyzed as an internal control. The data are shown as the mean ± S.D (n=2). Statistically significant differences are indicated (*, P<0.05; **, P<0.01; ***, P<0.001). (D) Immunoblotting and densitometric analysis of the expression of Yap and phosphorylated Yap (YapS127) in wild type ES cells and *Mst-/*- ES cells. Gapdh1 was analyzed as an internal control. The data are shown as the mean ± S.D (n=2). Statistically significant differences are indicated (*, P<0.05; **, P<0.01; ***, P<0.001).

To examine whether deletion of *Mst1* and *Mst2* affects the integrity of ES cells, we examined the expression profiles of pluripotency markers *Pou5f1*, *Nanog*, and *Sox2* by RT-PCR. There was an increase of *Nanog* transcripts in both *Mst-/-* ES cell lines, but the expression levels of *Pou5f1* and *Sox2* were similar in *Mst-/-* and wild type ES cells (Figure 2A). Immunofluorescence assay with antibodies against pluripotent markers Oct4 and SSEA1 revealed no significant difference between wild type ES cells and *Mst-/-* ES cells (Figure 2B). Further checking of the protein level of ES cell transcription factors, Nanog and Oct4, confirmed that Oct4 protein level was not affected by *Mst1/Mst2* deletion, but Nanog protein was higher in *Mst-/-* ES cells than wild type ES cells (Figure 2C). This result suggests that the expression of *Nanog* may be regulated by Mst1/Mst2 kinases. 

As a downstream effector of the Hippo pathway, Yap is also a pluripotent factor in ES cells and expression of *Yap* promotes reprogramming efficiency of mouse iPS cells. We therefore examined the expression of *Yap* by RT-PCR. The expression of *Yap* is similar in wild type ES cells and *Mst-/-* ES cells (Figure S2A). Further examination of Yap and phosphorylated Yap by western blot and immunofluorescence stain revealed that total Yap is not changed in *Mst-/-* ES cells, but phosphorylated Yap is significantly reduced in *Mst-/-* ES cells (Figure 2D and S2B). This observation is consistent with previous reports that Mst1/Mst2 phosphorylate Lats1/Lats2, which in turn phosphorylate Yap. As unphosphorylated Yap actively promotes cell proliferation, these data confirmed that the Hippo pathway, which is active in ES cells, is significantly downregulated in *Mst-/-* ES cells. As the effect of upregulation of active Yap, *Ctgf* and *Cyr61*, the downstream targets of Yap [38], were significantly increased in *Mst-/-* ES cells (Figure S2C). 

### Differentiation resistance of mouse Mst-/- embryonic stem cells

**Figure 3:**
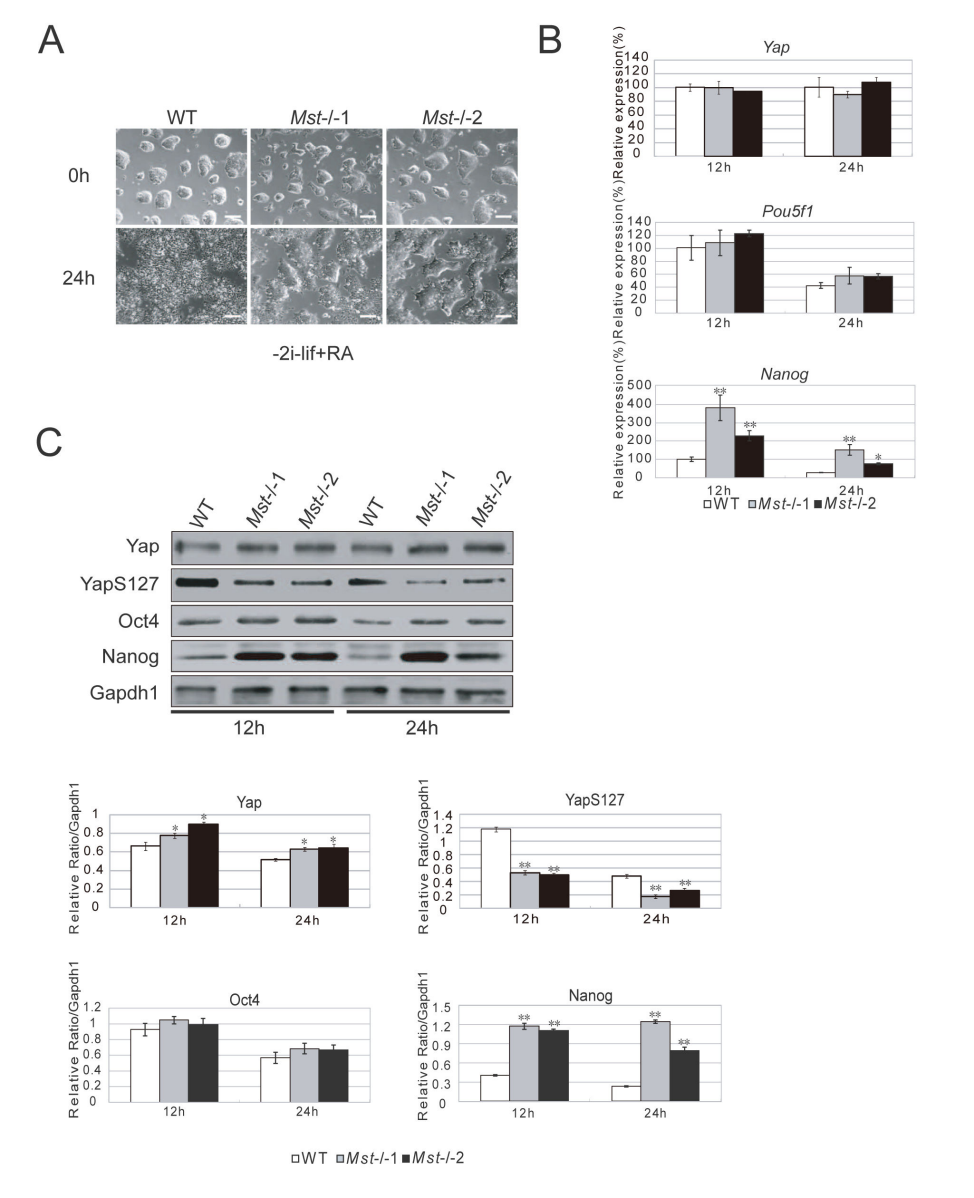
Differentiation resistance of *Mst-/-* ES cells. (A) Morphology of wild type ES cells and *Mst-/*- ES cells initially and 24 hour after growing in ES cell differentiation medium supplemented with RA, but not 2i and LIF. Scale bar, 200 μm. (B) Quantitative real-time PCR to examine the mRNA level of *Yap*, *Pou5f1* and *Nanog* in wild type ES cells and *Mst-/*- ES cells during ES cell differentiation medium for 12 hours and 24 hours. *Actin* was analyzed as an internal control. The data are shown as the mean ± S.D (n=3). Statistically significant differences are indicated (*, P<0.05; **, P<0.01; ***, P<0.001). (C) Immunoblotting and densitometric analysis of Yap, YapS127, Oct4 and Nanog in wild type ES cells and *Mst-/*- ES cells in ES cell differentiation medium for 12 hours and 24 hours. Gapdh1 was analyzed as an internal loading control. The data are shown as the mean ± S.D (n=2). Statistically significant differences are indicated (*, P<0.05; **, P<0.01; ***, P<0.001).

Based on above observations, we examined whether the maintenance of pluripotency was altered in ES cells by *Mst1/Mst2* deletion. We withdrew the molecular chemicals 2i (CHIR99021 and PD0325901) and LIF from the ES cell culture medium, and added retinoic acid (RA) to promote ES cell differentiation. We found that wild type ES cells completely lost colony morphology and were differentiated after 24 hours, while *Mst-/-* ES cells still maintained a certain level of colony morphology (Figure 3A). RT-PCR examination of the pluripotent markers showed that the mRNA level of *Nanog* was significant higher in *Mst-/-* ES cells than wild type ES cells, while the mRNA level of *Yap* and *Pou5f1* in *Mst-/-* ES cells and wild type ES cells was similar (Figure 3B). We further measured the protein level by western blot. *Mst-/-* ES cells expressed slightly more Yap protein than wild type ES cells from 12 hours after LIF withdrawal, but phosphorylated Yap (S127) was significantly decreased in *Mst-/-* ES cells compared to wild type ES cells, suggesting an increase of active unphosphorylated Yap in *Mst-/-* ES cells. Consistent with the upregulation of *Nanog* transcript, Nanog protein was also significantly increased in *Mst-/-* ES cells. And there was no obvious change of Oct4 between *Mst-/-* ES cells and wild type ES cells (Figure 3C). Taken together, these data showed that deletion of *Mst1* and *Mst2* increased the barrier of ES cell differentiation through upregulating the pluripotent marker unphosphorylated Yap and Nanog.

### Enhanced cell proliferation of mouse Mst-/- embryonic stem cells

**Figure 4:**
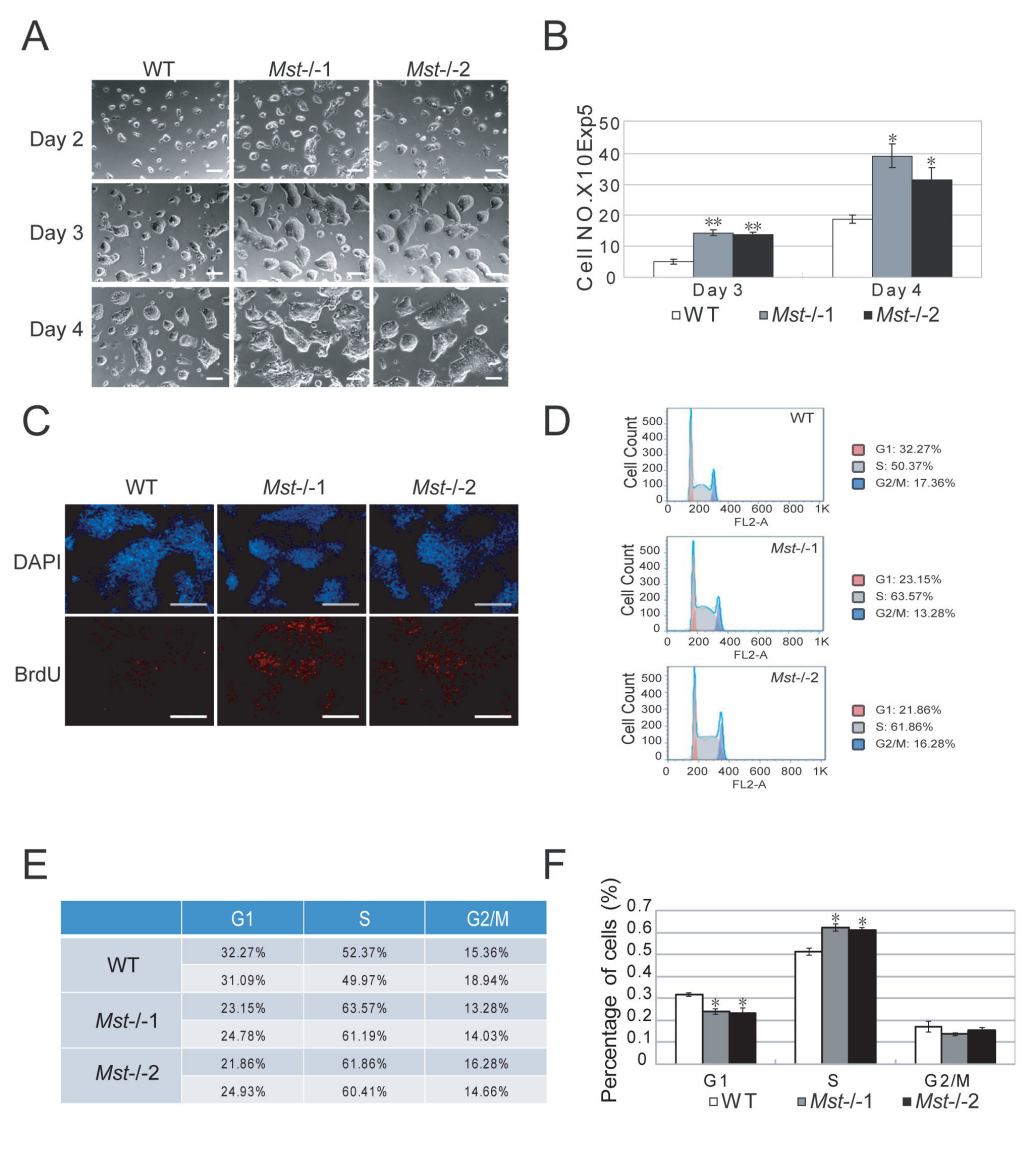
*Mst-/-* ES cells proliferate faster than wild type ES cells. (A) Morphology of 1x10^5^ wild type ES cells or *Mst-/*- ES cells grown in 2i+LIF ES medium for 2 days, 3 days and 4 days respectively. Scale bar, 200 μm. (B) Statistical analysis of the growth rate of wild type ES cells and *Mst-/*- ES cells on day 3 and day 4 culture. The data were shown as the mean ± S.D (n=3). Statistically significant differences are indicated (*, P<0.05; **, P<0.01; ***, P<0.001). (C) Immunofluorescence staining with BrdU antibodies to examine BrdU integration in wild type ES cells and *Mst-/*- ES cells after serum starvation for 12 hours. Cells are pulsed labeled with BrdU for 45 minutes. The nuclei were stained with DAPI. Scale bar, 200 μm. (D) Representative histograms of cell cycle distribution in *Mst-/*- ES cells and wild type ES cells. (E) Table of the cell cycle distribution in *Mst-/*- ES cells and wild type ES cells from two independent experiments. (F) Statistical analysis of cell cycle distribution in *Mst-/*- ES cells and wild type ES cells from two independent experiments. (*, P<0.05).

To assess the effects of Mst1 and Mst2 on ES cell proliferation, the same number of wild type and *Mst-/-* ES cells were trypsinized to single cells and plated. After 2 days, dome-shaped colonies were formed from single cells of both cell types. Interestingly, *Mst-/-* ES cell colony size was generally bigger than wild type ES cells (Figure 4A). Meanwhile, the cell numbers of *Mst-/-* ES cells in day 3 and day 4 cultures were significantly greater than wild type ES cells, suggesting *Mst-/-* ES cells have a higher proliferation capacity than wild type ES cells (Figure 4B). We then measured the expression of cell cycle related genes *Ccnd2* and *Ccnd3* by RT-PCR and found that *Mst-/-* ES cells expressed significantly higher *Ccnd2* and *Ccnd3* than wild type ES cells (Figure S6A).To further substantiate our observation, wild type and *Mst-/-* ES cells were pulse incorporated with BrdU for 1 hour after serum starvation and quantified by immunofluorescence staining and flow cytometry respectively. We observed significantly fewer BrdU-positive cells in the wild type ES cells, compared to the *Mst-/-* ES cells (Figure 4C, S6B and S6C). Cell cycle analysis by Propidium Iodide staining revealed that more than 60% of *Mst-/-* ES cells were at S phase, while about 50% wild type ES cell were at S phase (Figure 4D, 4E and 4F), suggesting that DNA is more actively synthesized in *Mst-/-* ES cells than wild type ES cells. 

### Defects of mouse Mst-/- embryonic stem cells in the formation of teratomas

Mice with *Mst1* or *Mst2* single gene knockout were viable and fertile, whilst *Mst1/Mst2* double knockout mice died early in gestation, suggesting that Mst kinases are essential for the early developmental program. 

To examine whether *Mst-/-* ES cells maintain the properties of ICM *in vivo*, we labeled the *Mst-/-* ES cells with GFP by lentivirus and injected them into 8-cell embryos. All *Mst-/-* ES cells successfully integrated into the inner cell mass (ICM), indicating that *Mst* deletion doesn’t affect cell surface identity at the ES cell state (Figure S3A). 

Then we injected wild type ES cells and *Mst-/-* ES cells subcutaneously into nude mice to check for teratoma formation. Wild type ES cells could form teratoma with tissue from all three germ layers within 6 weeks, but no tumor tissue was detected 6 weeks after *Mst-/-* ES cells were subcutaneously injected into nude mice. Even 8 weeks later, still no tumor was formed in *Mst-/-* ES cell injected mice (Figure S3B). RT-PCR examination of ES cells and teratoma revealed that the expression of *Yap*, *Mst1* and *Mst2* were higher in teratoma than ES cells (Figure S3C), suggesting that Hippo pathway may play an important role in teratoma formation.

To explore the reason why *Mst-/-* ES cells cannot form teratoma, we compared the expression of genes involved in apoptosis and tumorigenesis between wild type EBs and *Mst-/-* EBs by microarray experiment. *FoxO* family genes *FoxO1*, *FoxO3* and *FoxO4*, were slightly increased in *Mst-/-* EBs (Figure S3D). However, not all apoptosis related genes were upregulated. Some pro-apoptotic genes, such as *Bid* and *Bax* were increased in *Mst-/-* EBs; while other pro-apoptosis genes such as *Bmf* and *Bcl2112* were downregulated in *Mst-/-* EBs. Similarly, some anti-apoptosis genes such as *Bcl2l1* and *Birc5* were increased in *Mst-/-* EBs, while another anti-apoptosis gene *Bcl2* was decreased. Although it was reported that greater number of apoptotic cells were detected in *Mst1/Mst2* double knockout embryos than *Mst1/Mst2* single copy gene knockout embryos during E8.5 to E9.5[13], it was not for sure whether apoptosis were enhanced after ES cells were injected for teratoma. Interestingly, some tumorigenesis related genes *Afp*, *CD34*, *Eno2* and *Nes* were obviously reduced in *Mst-/-* EB, suggesting a reduced tumorigenesis capacity during *Mst-/-* ES cell differentiation. This may be one of the reasons that *Mst-/-* ES cell cannot form teratoma. 

### Deletion of *Mst1/Mst2* distorts embryonic stem cell differentiation *in vitro*

**Figure 5:**
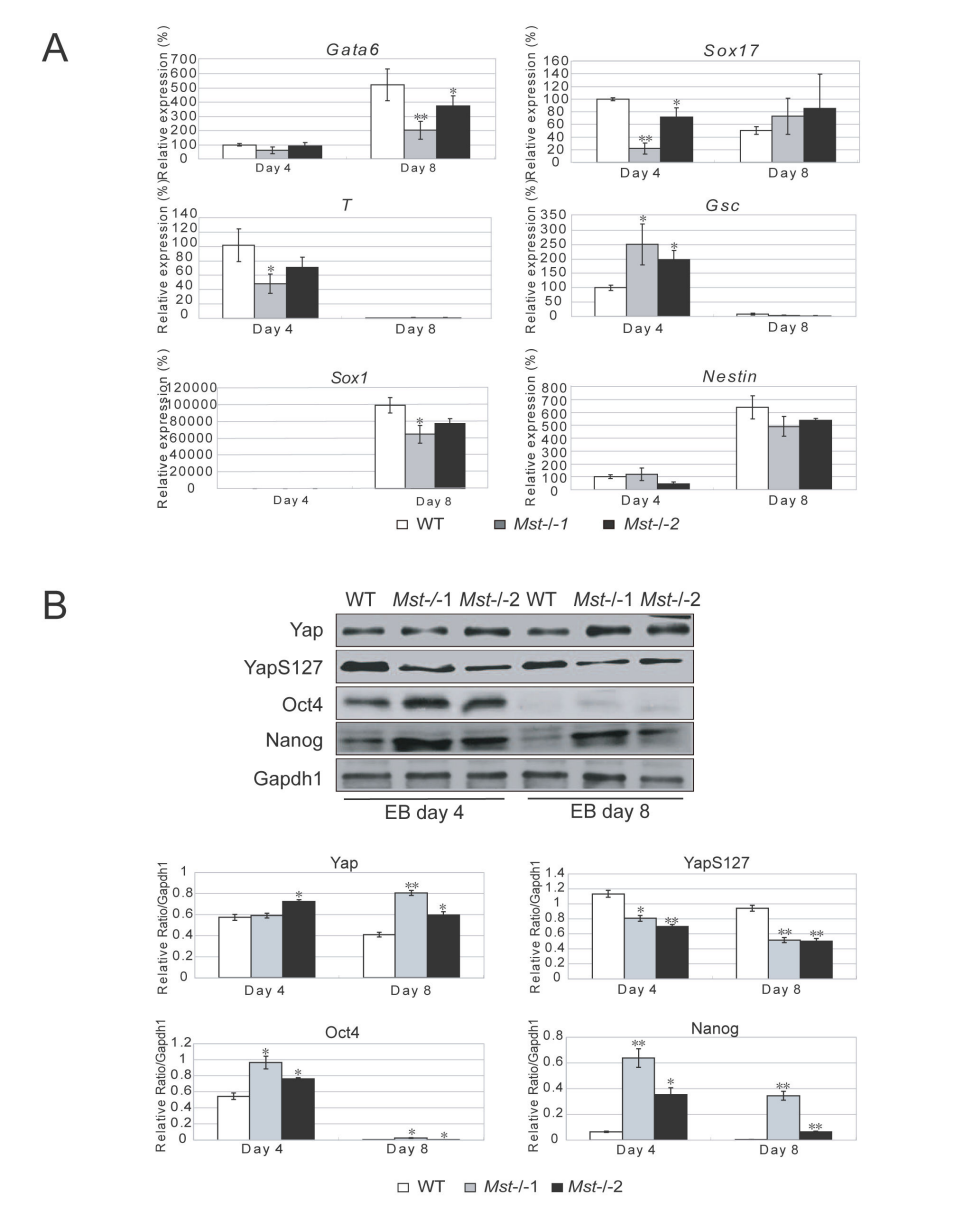
Depletion of *Mst1/Mst2* affects proper EB differentiation. (A) Quantitative real time PCR to reveal the mRNA level of endoderm markers *Gata6* and *Sox17*, mesoderm markers *T* and *Gcs*, and ectoderm markers *Sox1* and *Nestin* in wild type EBs and *Mst-/*- EBs at day 4 and day 8 during EB formation. *Actin* was analyzed as an internal control. The data were shown as the mean ± S.D (n=3). Statistically significant differences are indicated (*, P<0.05; **, P<0.01; ***, P<0.001). (B) Immunoblotting and densitometric analysis to check the protein level of Yap, YapS127, Oct4 and Nanog in day 4 and day 8 wild type EBs and *Mst-/*- EBs. Gapdh1 was analyzed as an internal control. The data are shown as the mean ± S.D (n=2). Statistically significant differences are indicated (*, P<0.05; **, P<0.01; ***, P<0.001).

To further examine the effect of depletion of *Mst1/Mst2* in ES cell differentiation, we examined the expression of multiple lineage markers in wild type ES cells and *Mst-/-* ES cells at both day 4 and day 8 during EB formation by RT-PCR. As shown in Figure 5A, endodermal markers, *Gata6* and *Sox17*, were affected in *Mst-/-* EBs. Compared to the wild type EBs the *Sox17* level was significantly lower in day 4 *Mst-/-* EBs, whilst the *Gata6* level was lower in day 8 *Mst-/-* EBs. The expression of ectodermal markers *Sox1* and *Nestin* and mesoderm markers *T* and *Gsc* were also slightly disturbed in *Mst-/-* EBs (Figure 5A). We also selectively examined protein level of three germ layer markers by western blot. Ectoderm marker Pax6 was increased in day 4 *Mst-/-* EBs and mesoderm marker T was upregulated in day 8 *Mst-/-* EBs, while endoderm marker Gata6 was decreased in day 8 *Mst-/-* EB (Figure S4A). To find out what other genes are affected by *Mst* deletion during EB formation, expression profile of day 4 and day 8 *Mst-/-* EBs and wild type EBs were compared by microarray experiment. In keeping with the quantitative RT-PCR results, microarray data showed that the expression of multiple pluripotency-related genes and lineage associated genes was distorted. Multiple pluripotent markers, such as *Dppa2*, *Dppa3*, *Dppa4*, *Oct4*, *Sox2*, *Nanog*, *Esrrb*, *Tcl1*, *Lefty1* and *Fgf4* were higher in *Mst-/-* EBs than wild type EBs (Figure S4B). Many Ectoderm markers *Tubb3*, *Fgf8*, *Notch1* and *Fgf5* were also significantly higher in *Mst-/-* EBs than wild type EBs; whilst endoderm markers *Gata6*, *Gata4, Cxcr4* and *Sox17*, were significantly lower in day4 *Mst-/-* EBs than wild type EBs. Mesoderm markers *T* and *Mixl1* were similarly expressed in day 4 *Mst-/-* EBs and wild type EBs, but higher in day 8 *Mst-/-* EBs. 

To further investigate the differences between *Mst-/-* EBs and wild type EBs, we also measured the protein levels of Yap, Oct4 and Nanog during EB formation at day 4 and day 8. An evidently higher protein level of Oct4 and Nanog was detected in day 4 *Mst-/-* EBs compared to wild type EBs. At day 8, both Oct4 and Nanog were undetectable in wild type EBs, but Nanog was still expressed in *Mst-/-* EBs (Figure 5B), whilst inactivated phosphorylated Yap was consistently expressed at a lower level in *Mst-/-* EBs than wild type EBs at both day 4 and day 8. There was no obvious difference in the total Yap protein level between *Mst-/-* EBs and wild type EBs at day 4, but Yap protein level was higher in *Mst-/-* EBs than wild type EBs at day 8. Therefore, unphosphorylated active Yap was higher in *Mst-/-* EBs than in wild type EBs. Taken together, pluripotent markers Oct4, Nanog and active Yap were expressed at a higher level in *Mst-/-* EBs than wild type EBs, indicating that *Mst-/-* ES cells display differentiation resistance during EB formation (Figure 5B). This finding demonstrates that *Mst-/-* ES cells show distorted early germ layer marker expression, which may be due to sustained higher expression of pluripotent markers during *Mst-/-* EB formation. 

### *Mst1/Mst2* are required for embryonic stem cells to differentiate into cardiac progenitor cells

Besides studying the role of Mst1/Mst2 in early germ layer formation, we also examined the potential of *Mst-/-* ES cells at later lineage cell differentiation. To generate neural progenitor cells, day 4 wild type and *Mst-/-* EBs were attached to tissue culture plates in N2 medium. At day 2, epithelium-like cells were formed in both cell lines. And at day 8, neurosphere-like colonies were observed both in wild type and *Mst-/-* cells. These cells could be passaged on 0.01% Poly-L-ornithine coated culture dishes and showed proliferation in VEGF and BFGF medium (Figure S5A). The identity of NSCs was further confirmed through immunostaining with antibody against the NSC marker *Pax6* (Figure S5B). Microarray analysis also revealed that many neurongenesis related genes such as *Mapt, Nrsn1*, *Neurod1* and *Nefm*, were expressed at a higher level in *Mst-/-* EBs than wild type EBs at day 4, indicating that Mst1/Mst2 may involve in repressing ES cell to neural lineage differentiation. Meanwhile, the expression of skin development related genes was similar in wild type EBs and *Mst-/-* EBs, suggesting the repressive role of Mst1/Mst2 in later ectoderm tissue differentiation is specific (Figure S5C).

Next, we compared the expression of some later endoderm tissue markers in *Mst-/-* EBs and wild type EBs. Although the expression of some genes is disturbed due to *Mst1/Mst2* depletion, there is no tissue specific pattern change (Figure S5C). Liver specific markers Alb and AAT protein are at the similar level in wild type EBs and *Mst-/-* EBs (Figure S5D). 

Meanwhile, we also examined *Mst-/-* ES cells for later mesoderm tissue differentiation potential. Skeletal muscle associated gene showed no changes between *Mst-/-* EBs and wild type EBs (Figure S7A). Although the expression level of some genes related to smooth muscle, endothelial cells and mesenchymal stem cells (MSCs) was different between *Mst-/-* EBs and wild type EBs, no lineage specific expression change pattern was observed. Interestingly, multiple hematopoietic stem cell (HSC) associated genes such as *CD34*, *CD38*, *Esam* and *Ngfr* were significantly lower in day 8 *Mst-/-* EBs than wild type EBs, suggesting a developmental delay of this lineage. Besides, cardiac stem cell genes *Tbx5*, *Smarcd3*, *Isl1*, *Mesp1*, *Kdr*, *Etv2* and *Nkx2.5* were dramatically decreased in day 8 *Mst-/-* EBs. Cardiomyocyte related genes, such as *Igf1*, *Ankrd1*, *Fli1* and *Myh6* were also downregulated in day 8 *Mst-/-* EBs. Representative genes of later mesoderm tissues were also selected to validate by RT-PCR, the results matched the microarray profile and also confirmed the downregulation of cardiac lineage genes in *Mst-/-* EBs (Figure S7B). 

**Figure 6:**
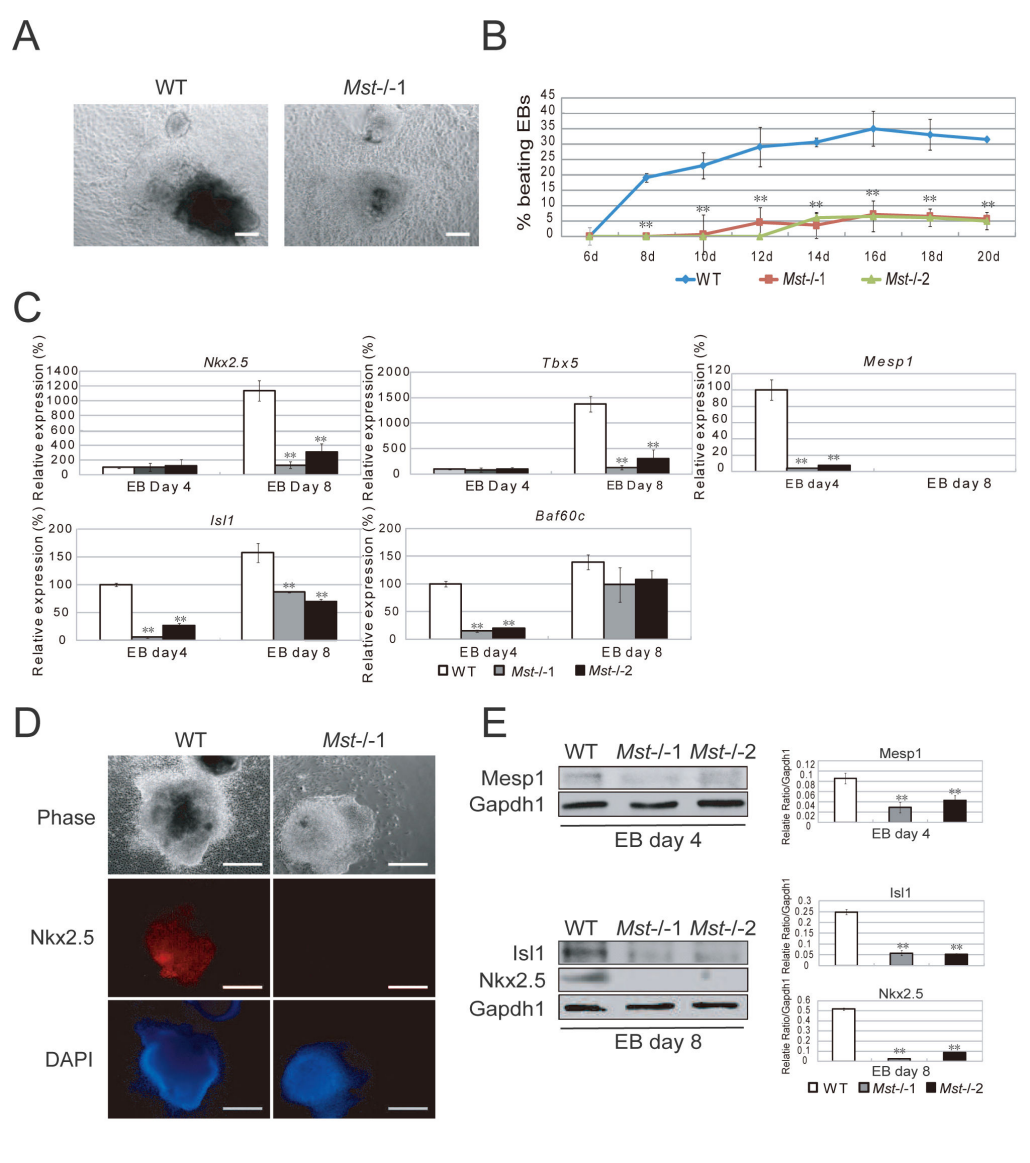
ES cell to cardiac progenitor cell differentiation is disturbed by *Mst1/Mst2* depletion. (A) Phase contrast pictures of differentiated wild type EBs and *Mst-/*- EBs in cardiac differentiation medium. Scale bar, 200 μm. (B) Percentage of spontaneously beating EBs determined from day 6 to day 20 during differentiation (n>100 per time point). *Mst-/-*EBs showed a significant less beating EBs than wild type EBs. Experiments were performed in triplicate, and error bars represent SD. Statistically significant differences are indicated (*, P<0.05; **, P<0.01; ***, P<0.001). (C) Relative mRNA levels of cardiac progenitor cell markers *Nkx2.5, Tbx5, Mesp1, Isl1* and *Baf60c* in wild type and *Mst-/*- EBs at day 4 and day 8 during EB formation. *Actin* was used as an internal control. The data are shown as the mean ± S.D (n=3). Statistically significant differences are indicated (*, P<0.05; **, P<0.01; ***, P<0.001). (D) Immunofluorescence stain with antibody against Nkx2.5 to examine cardiac progenitor marker *Nkx2.5* expression in the wild type EBs and *Mst-/*- EBs in cardiac differentiation medium for 8 days. Scale bar, 200 μm. (E) Immunoblotting and denstitometric analysis with antibody against Mesp1, Isl1 and Nkx2.5 to check their expression in wild type EBs and *Mst-/*- EBs in cardiac differentiation medium for 4 days or 8 days. Gapdh1 was analyzed as an internal control. The data are shown as the mean ± S.D (n=2). Statistically significant differences are indicated (*, P<0.05; **, P<0.01; ***, P<0.001).

To validate whether *Mst-/-* ES cells have defect in cardiac lineage differentiation, day 6 wild type and *Mst-/-* EBs were plated on human fibronectin treated dishes to induce cardiac lineage cells. On day 3, a lot of beating cell clumps were observed in wild type sample but no beating cell clumps were observed in *Mst-/-* samples (Figure 6A, Movie S1 and Movie S2). Longer culture time revealed that a few beating cell clumps emerged from day 12 in *Mst-/-* samples. However, the beating cell clumps in *Mst-/-* samples were about 6 times less than wild type EBs even after culturing for 20 days (Figure 6B). RT-PCR also confirmed that cardiac progenitor markers *Nkx2.5*, *Tbx5*, *Mesp1*, *Isl1* and *Baf60c* were significantly repressed in *Mst-/-* samples as compared to wild type samples (Figure 6C). Immunofluorescence revealed that Nkx2.5 was expressed in the wild type samples but not in the *Mst-/-* samples (Figure 6D). Western blot analysis of the protein lysates also confirmed expression of Mesp1, Isl1 and Nkx2.5 in the wild type samples, but not in the *Mst-/-* samples (Figure 6E). All these data demonstrated that Mst1 and Mst2 were required for ES cells to differentiate into cardiac lineage cells.

### *Mst1/Mst2* are involved in cardiogenesis through regulating non-canonical wnt ligands

Wnt signaling plays an important role in regulating cardiac progenitor cell specification. Canonical Wnt3a/β-catenin signaling is important for cardiomyogenesis during development. While non-canonical Wnt ligand Wnt2 accelerates cardiac myocyte differentiation from ES-cell derived mesodermal cells and Wnt5a is essential for second heart field progenitor development. 

**Figure 7:**
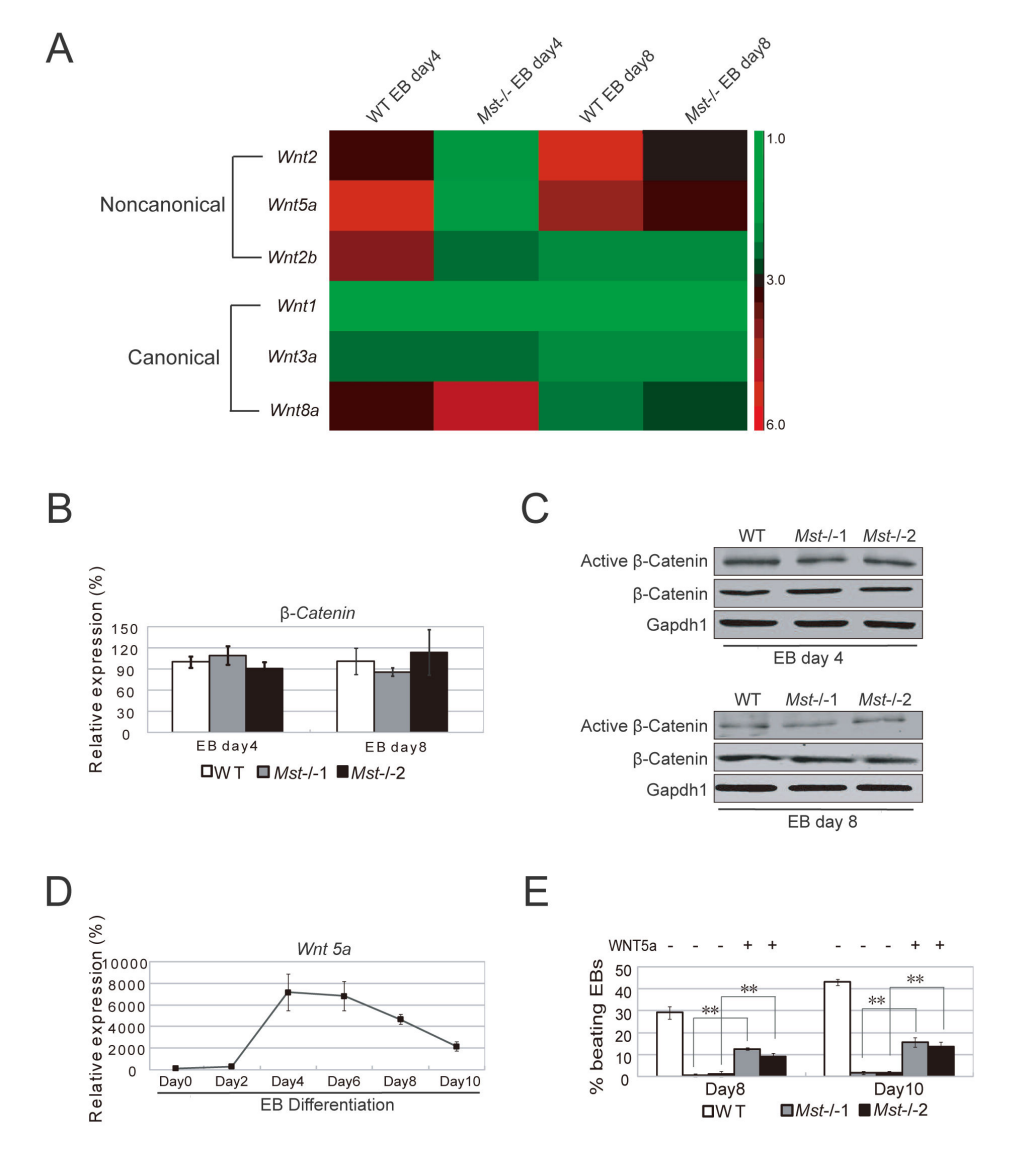
ES cell to cardiac progenitor cell differentiation is disturbed by *Mst1/Mst2* depletion. (A) Heatmap of the expression of non-canonical Wnt signaling ligands (Wnt2, Wnt2b and Wnt5a) and canonical Wnt ligands (Wnt1, Wnt3a, Wnt8a and Wnt11) in day 4 and day 8 wild type EBs and *Mst-/*- EBs. (B) Relative mRNA levels of β-catenin in wild type and *Mst-/*- EBs at day 4 and day 8 during EB formation. *Actin* was used as an internal control. The data are shown as the mean ± S.D (n=3). Statistically significant differences are indicated (*, P<0.05; **, P<0.01; ***, P<0.001). (C) Immunoblotting analysis with antibodies against Active β-catenin and total β-catenin to check its expression in day 4 and day 8 wild type EBs and *Mst-/*- EBs. Gapdh1 was analyzed as an internal control. (D) Relative mRNA levels of Wnt5a during EB formation from day0 to day10. *Actin* was used as an internal control. The data are shown as the mean ± S.D (n=3). Statistically significant differences are indicated (*, P<0.05; **, P<0.01; ***, P<0.001). (E) Recombinant Wnt5 were supplemented to the *Mst-/-*EB culture from day 2 and day 10. Wild type EBs and *Mst-/*- EBs were grown in non-Wnt5a supplemented medium as controls. The percentage of beating EBs was profiled on day 8 and day 10 after initiating EBs culture. The data are shown as the mean ± S.D (n=3). Statistically significant differences are indicated (*, P<0.05; **, P<0.01; ***, P<0.001).

To check whether there is any link between the defect of *Mst-/-* ES cell cardiac lineage differentiation and the Wnt signaling abnormal regulation, we examined the expression of Wnt ligand genes in day 4 and day 8 wild type and *Mst-/-* EBs. The expression of canonical Wnt ligand gene *Wnt1 and Wnt3a* showed no change between in *Mst-/-* EBs and wild type EBs, while another canonical Wnt ligand gene *Wnt8a* was slightly increased (Figure 7A). RT-PCR and western blot to examine their downstream mediator β-catenin revealed that total and active β-catenin were at the similar level in wild type EBs and *Mst-/-* EBs, indicating the defect of *Mst-/-* ES cell to cardiac lineage differentiation may not be related to canonical Wnt signaling (Figure 7B and 7C). On the other hand, several non-canonical Wnt ligand genes, such as *Wnt2*, *Wnt2b*, and *Wnt5a*, were significantly downregulated in *Mst-/-* EBs (Figure 7A). Exogenous Wnt2 can enhance ES cell to cardiomyocyte differentiation. To validate whether Wnt5a can also enhance the differentiation, we first examined Wnt5a expression during wild type EB differentiation. We found that Wnt5a was dramatically increased in day 4 EBs (Figure 7D). Therefore we added Wnt5a recombinant protein to day 2 *Mst-/-* EB culture. Notably, *Mst-/-* EBs grown in medium supplemented with Wnt5a showed increase number of beating EBs compared to *Mst-/-* EBs grown in non-Wnt5a medium on day 8 and day 10, although less than wild type EBs, indicating that Wnt5a can partially rescue *Mst-/-* EB defect for cardiomyocyte differentiation (Figure 6B and 7E). Taken together, these data suggest that Mst1/Mst2 may involve in cardiomyocyte differentiation through crosstalking with non-canonical Wnt signaling.

## Discussion

Mst1 and Mst2, the key components of the Hippo pathway in mammals, have been widely studied in different cell types, such as liver, neural, heart and intestine cells, in humans and mice. In these cell types, *Mst1* and *Mst2* are mainly involved in restricting cell proliferation, promoting apoptosis and regulating tumorigenesis [12,20,22-24,39]. Mst1 and Mst2 also play critical roles in mouse embryo development. Single *Mst1* or *Mst2* deletion gives rise to only minor defects in mice after birth, but deletion of both *Mst1* and *Mst2* simultaneously leads to embryonic lethality at E8.5 as a result of obvious defects in placental development and vascular patterning, etc [11-13]. 

To dissect the role of Mst1/Mst2 in mouse embryonic stem cells, we established *Mst-/-* ES cells. We found that phosphorylated Yap was decreased whilst unphosphorylated Yap was increased in *Mst-/-* ES cells. Consistently, Yap downstream targets, *Ctrf* and *Cyr61* were also significantly upregulated in *Mst-/-* ES cells. This confirms that the Hippo pathway is active in mouse ES cells and that Mst1/Mst2 contributes to deactivate Yap. But it is interesting that residual phosphorylation of Yap can be detected in *Mst-/-* ES cells, indicating that Mst kinases may not be the exclusive components that lead to Yap inactivation and alternative pathways may exist to inactivate Yap. Actually, it was reported that Akt kinase can phosphorylate Yap at Serine 127 to regulate Yap translocation [40,41]. Besides, angiomotin (Amot) and angiomoti-like1 (AmotL1) physically interact with Yap and restrict Yap activity in a Hippo pathway-independent manner [42,43]. Therefore, Yap regulation *in vivo* is a multi-level complicated procedure. Unphosphorylated Yap is an important pluripotent factor which plays a critical role in maintaining embryonic stem cell pluripotency and improves the efficiency of iPSC formation. Yap directly binds to the regulatory regions of *Nanog* in mouse ES cells based on the ChIP-seq data published [26]. We consistently detected higher level of *Nanog* in *Mst-/-* ES cells and early *Mst-/-* EB than wild type control, suggesting that unphosphorylated Yap may directly binds to *Nanog* and activate its expression. Since ES cells with high level of Nanog show differentiation resistance, *Mst-/-* ES cells also show differentiation resistance due to upregulation of Nanog.

It is very intriguing that *Mst-/-* Es cells show enhanced cell proliferation (Figure 4, S6A and S6B), but cannot form teratomas in nude mice. *Mst-/-* ES cells obviously maintained ES cell surface identity, as *Mst-/-* ES cells can integrate into ICMs (Figure S4). But *Mst-/-* EB differentiation capacity is somehow distorted and weakened. For example, *Mst-/-* ES cells show great defect in cardiac lineage differentiation. It is unclear whether the differentiation distortion of ES cells links to their teratoma formation capacity. As embryonic carcinoma cells (EC cells) are also pluripotent, but with lower differentiation capacity as compared to ES cells, they can form teratocarcinomas, a malignant teratoma. Although *Mst-/-* embryos show greater number of cell apoptosis than the control [13]. Our *Mst-/-* EB microarray data show no strong evidence of enhance apoptosis during differentiation. Whereas relative lower expression level of tumorigenesis genes *Eno2*, *Nes*, *CD34* and *Afp* in *Mst-/-* EBs than wild type EBs may be one of the reasons that *Mst-/-* ES cells cannot grow teratoma. Besides, it is reported that natural killer (NK) cells which are still active in nude mice preferentially involved in rejecting undifferentiated ES cells [44]. *Mst-/-* ES cells show differentiation resistance. Hence, they may be more susceptible for elimination by NK cells than wild type ES cells in nude mice. This may account for another reason for the failure of teratoma formation of *Mst-/-* ES cells. Above all, the inability of ES cells to form teratomas after *Mst1/Mst2* deletion is quite interesting. It indicates that we can prevent teratoma formation from ES cells through inhibition of Mst1/Mst2 kinase activities, though further investigations are required to elucidate the mechanism of this. Although it is premature to translate this idea into an application, the transplantation of ES cells, combined with Mst kinase inhibition, may be worthy of trials in the repair of certain lineage cells, such as neural progenitor cells, in which *Mst1/Mst2* do not play essential roles. Such treatments will however be limited by the fact that Mst1/Mst2 are required for differentiation into cardiac progenitor cells and possibly other cell lineages.

To dissect the role of Mst1/Mst2 in ES cell differentiation, in this study, we systematically compared the transcriptome between *Mst-/-* ES cells and wild type ES cells during their EB formation. We found that *Mst-/-* ES cells display some lineage differentiation distortion. For example, the expression of early endoderm gene is obviously decreased in day 4 *Mst-/-* EBs, while the expression of most early ectoderm genes is upregulated, suggesting Hippo signaling may play an important role in early germ layer determination. There is no obvious disturbance in early mesoderm formation in *Mst-/-* EBs. But Microarray comparison of the mesoderm derived tissue expression profile revealed that markers of cardiac lineage and hematopoietic stem cells (HSC) are synergistically affected, suggesting an important role of *Mst1/Mst2* in the differentiation of mesoderm to these lineages. 

*Mst1/Mst2* double knockout mice can form heart *in vivo*, however, the organization and morphology were abnormal, suggesting Mst1/Mst2 play an essential role in heart formation. Recent research reported crosstalk between the Hippo pathway and canonical Wnt signaling in regulating cardiomyocyte proliferation [45-47]. In order to gain insight into the detailed mechanism, we analyzed the microarray data of day 4 and 8 EBs and found that Wnt ligands show different expression between *Mst-/-* EBs and wild type EBs. As canonical Wnt signaling downstream mediator β-catenin show no obvious change in *Mst-/-* EBs, canonical Wnt signaling therefore is not responsible for the cardiac lineage differentiation defect in the *Mst-/-* ES cells. Interestingly, non-canonical Wnt signaling ligands *Wnt2*, *Wnt2b* and *Wnt5a* were significantly decreased in *Mst-/-* EBs compared to wild type cells. *Wnt2b* null ES cells form no beating EBs, with much lower levels of cardiac genes than wild type EBs [48]. Exogenous Wnt2 can promote ES cell to cardiac lineage cell differentiation [49]. The *Wnt5a* signal is instructive for the differentiation of cardiac neural crest (CNC) cells during formation of the aortopulmonary septum through a non-canonical Wnt/Ca2^+^ pathway and hence essential for second heart field progenitor development [50-53]. We found that addition of Wnt5a recombinant proteins can partially rescue cardiac differentiation defect of *Mst-/-* EBs, which further substantiate that Hippo pathway regulates cardiac lineage differentiation through crosstalk with non-canonical Wnt signaling. Our studies indicate however that there might be connections between these two pathways. This area requires further exploration.

## Materials and Methods

### ES cell derivation and cell culture

All mouse experiments are performed under the approval from Animal Experimentation Ethics Committee (AEEC) in the Chinese University of Hong Kong. *Mst-/-* knockout mouse embryos were obtained by crossing *Mst1+/-, Mst2-/-* (C57BL/6) female and male mice. E3.5 blastocysts were ﬂushed out from the uterus and cultured on mitomycin treated MEF feeder in a 96-well plate with N2B27 medium with 2i (PD0325901, 0.4mM: Stemgent, San Diego, CA; CHIR99021, 3mM: Stemgent) and LIF (1000 U/ml). The ICM outgrowths were treated with 0.05% Trypsin (Invitrogen) and passaged on a 24-well plate until stable ES cell lines were obtained. The ES cells were maintained on feeders under the normal ES medium (DMEM supplemented with 15% FBS, 0.1 mM non-essential amino acids, 0.1mM 2-mercaptoethanol, 2mM Glutamine, 100U/ml penicillin/streptomycin and 1000U/ml LIF). To obtain feeder free ES cells, the ES cells were grown on a 0.2% Gelatin coated dish in 2i+LIF medium. For ES cell differentiation, 2i and LIF were removed from the ES medium and 2μM RA was added to enhance differentiation. For EB formation, wild type ES cells and *Mst-/-* ES cells were trypsinized into single cells and then replaced at 1x10^6^ cells in a 10 cm non-adherent dish in ES cell medium devoid of 2i and LIF. Floating EBs were harvested at 4 days or 8 days for analysis. For cell proliferation assay, 1x10^5^ cells were plated in 6-well dishes. The cells were trypsinized to single cells and counted on day 3 and day 4. For neural stem cell differentiation and cardiac progenitor cell differentiation, previously described methods were followed [54,55]. Briefly, for neural stem cell differentiation, day 4 EBs were cultured in DMEM+10%FBS medium for 24 hours on tissue culture plate to allow EBs to attach to the plate. The medium was subsequently switched to DMEM/F12 (1:1) supplemented with N2 and changed every 2 days. For passage, cells were dissociated by 0.25% trypsin + 0.04% EDTA and then replated in DMEM/F12 (1:1) supplemented with N2 and bFGF (5ng/ml) medium. For cardiac progenitor cell differentiation, day 6 EBs were plated onto a tissue culture plate treated with human fibronectin for further analysis. For the rescue experiment, WNT5a recombinant protein were purchased form R&D company, 200ug/ml WNT5a were added to *Mst-/-* EB samples on day 2.

### Quantitative real-time PCR

Total RNAs were extracted from ESC or EB samples using Trizol reagent (Invitrogen) according to the manufacturer’s instructions. Aliquots of 1μg of RNA were used as templates for reverse transcription with the PrimeScript RT reagent Kit (TaKaRa) according to the instructions. Real-time PCR analysis was performed using the ABI Prism 7900HT machine (Applied Biosystems) with the SYBR Premix Ex Taq (TaKaRa). The generated threshold cycle (CT) value for each transcript was normalized against the CT value of an internal control, *Actin*, and subsequently normalized against the CT value of corresponding transcripts of the control sample. The sequences of RT primers are listed in Table S1. For each primer used, only one correct size band was observed. All experiments were repeated at least three times with samples from independent experiments. 

### Western blot analysis

ESC or EB samples were collected and washed twice with PBS. RIPA buffer (150 mM NaCl, 10 mM Tris, pH 7.2, 0.1% SDS, 1.0% Triton X-100, 1.0% deoxycholate, 5 mM EDTA, and protease inhibitors) was used to lyse the cells. Protein samples were separated by SDS-polyacrylamide gel electrophoresis and transferred to PVDF membranes (Pall corporation). The membranes were blocked with 5% nonfat dry milk (BD company) in TBST+0.1% Tween and incubated with primary antibody in TBST+0.1% Tween overnight at 4°C. The primary antibodies and dilutions used were mouse anti-Gapdh (A-3) (sc-137179; Santa Cruz Biotechnology) at 1/2,000, goat anti-Oct3/4 (N-19) (sc-8628; Santa Cruz Biotechnology) at 1/2,000, rabbit anti-Nanog (RCAB0002P-F; Cosmo bio co) at 1/2000, rabbit anti-Mst1 (catalog no. 3682; Cell Signaling Technologies) at 1/1,000, rabbit anti-Mst2 (catalog no. 3952; Cell Signaling Technologies) at 1/1,000, rabbit anti-Yap (catalog no. 4912; Cell Signaling Technologies) at 1/1,000, rabbit anti-Phospho-Yap (Ser127) (catalog no. 4911; Cell Signaling Technologies) at 1/1,000, rabbit anti-Pax6 (Ab2237; Milipore) at 1/1,000, goat anti-Brachyury (N-19) (sc-17743; Santa Cruz Biotechnology) at 1/1,000, rabbit anti-Gata6 (AF1700; R&D system) at 1/1,000, goat anti-Mesp1 (T-15) (sc-163078; Santa Cruz Biotechnology) at 1/1,000, goat anti-Nkx2.5 (N-19) (sc-8697; Santa Cruz Biotechnology) at 1/1,000, rabbit anti-Islet1 (T-15) (ab-20670; Abcam) at 1/1,000, mouse anti active-β-Catenin (05-665; Milipore) at 1/1,000, rabbit anti β-Catenin (95875; Cell signaling) at 1/1,000, goat anti-AAT (G-17) (sc-14586; Santa Cruz Biotechnology) at 1/1,000, goat anti-Albumin (A90-134A; Bethyl) at 1/1,000Signals were detected with ECL detection reagents (Abcam). Densitometric analysis was performed with software ImageJ. The relative ratio of protein dentisty was calculated by normalizing with Gapdh1. 

### Alkaline Phosphatase staining

ES cells were fixed with a fixative solution (90% Methanol and 10% Formaldehyde) for 20 minutes at room temperature, and then rinsed with 1x Rinse buffer (20mM Tris-HCl, pH 7.4, 0.15M NaCl, 0.05% Tween-20), followed by incubating the cells in Alkaline Phosphatase staining solution (Fast Red violet/Naphthol AS-BI phosphate solution/Water=2:1:3) in the dark at room temperature for 15 minutes. The cells were then washed with PBS buffer and photographed with an Olympus microscope (FV1000, Olympus, Tokyo, Japan).

### Immunofluorescence stain

Immunoﬂuorescence staining of ES and differentiated cells was performed with the following standard protocols. Cells were fixed in 4% paraformaldehyde at room temperature for 10 minutes and permeabilized with 0.5% Triton X-100 (for nuclear stain), followed by blocking with 1% BSA in PBS for 1 hour and then primary antibody overnight at 4°C. The primary antibodies and dilutions used were anti-SSEA1 antibody (SC-21702, Santa Cruz Biotechnology) at 1:200, goat anti-Oct3/4 (N-19) (sc-8628; Santa Cruz Biotechnology) at 1/200, rabbit anti-Yap (catalog no. 4912; Cell Signaling Technologies) at 1/100, rabbit anti-Phospho-Yap (Ser127) (catalog no. 4911; Cell Signaling Technologies) at 1/100, rabbit anti-Pax6 (Ab2237; Milipore) at 1/200, goat anti-Nkx2.5 (N-19) (sc-8697; Santa Cruz Biotechnology) at 1/200, After washing with PBS, the samples were incubated with the appropriate secondary antibodies, conjugated with Alexa Fluor 594 (Molecular Probes) in PBS, for 1 hour at room temperature. Nuclei were stained with 4, 6-diamidino-2-phenylindole dilactate (DAPI; Molecular Probes, D3571) for 10min. Images were captured with an Olympus fluorescence microscope (FV1000, Olympus, Tokyo, Japan). 

### Teratoma Assays

2x10^6^ wild type ES cells and *Mst-/-* ES cells respectively were harvested with 0.25% Trypsin, suspended in 0.9% NaCl and injected subcutaneously into nude mice. Six weeks after injection, the nude mice were euthanized and tumors removed were fixed in 4% paraform, and then subjected to hematoxylin and eosin stain for histological analysis.

### Microarray

Total RNA derived from wild type and *Mst-/-* ES cell and EB samples (day 4 and 8) was extracted with Trizol (Invitrogen) and purified with an RNeasy mini-kit (Qiagen). The purified RNAs were reversed-transcribed, labeled and hybridized to Affymetrix mouse exon 1.0 ST Array. The arrays were processed following the manufacturer’s instruction. The data were analyzed with software Partek and Genespring. The threshold for gene expression was set at >1.5-fold. Miscroarray data are accessible under GEO accession number GSE50219.

### Cell cycle analysis

Wild type ES cells and *Mst-/-* ES cells were trypsinized into single cells and then fixed in cold 70% ethanol overnight. RNA was removed by 100 µg/ml Rnase (Sigma) treatment at room temperature for 20 minutes. The cells were then stained with 5 mg/ml propidium iodide (PI, Sigma) at 37 degree for 1 hour. Flow cytometric analysis was carried out on 10,000 gated events using FACSCalubur (BD Biosciences). Then cell cycle phase distribution was analyzed using software Flowjo (version 7.6). 

### Brdu cell proliferation assay

Wild type and *Mst-/-* ES cells were pulse-labelled with BrdU (1:1000; Cell signaling) for 45 minutes. ES cells were dispersed into single cells; the cells were fixed overnight in 70% ethanol at 4 degree. DNA denaturation was subsequently performed by incubation in 1.5N HCl for 20 minutes at room temperature. The cells then washed and incubated with 0.1 M sodium tetraborate for 10 minutes at room temperature. The cells were incubated with Alexa Fluor 488- conjugated mouse anti-BrdU antibody (1:100; Molecular Probes) in 2% BSA-PBS for 2 hours at 4 degree. The cells were then incubated with 100 mg/ml RNase (Sigma) for 15 minutes and then subject to FACSCalibur (BD Biosciences) to record the data. The data was analysed by CellQuest program.

## Supporting Information

Figure S1 **Mst-/- ES cell derivation.** (A) 3.5 day blastocysts obtained by crossing Mst1+/-, Mst2-/- male and female mice. Scale bar, 200μm. (B) ICM outgrowth formed 5 days after a single blastocyst was seeded on MEF feeder in 2i+LIF ES medium. Scale bar, 200μm. (C) Schematics of targeted deletion loci (kinase domain) of Mst1 and Mst2. Boxes denote exons and lines denote intron.
(TIF)

Figure S2 **The expression of Yap and Yap targets in *Mst-/-* ES cells.** (A) Quantitative RT-PCR to check mRNA level of *Yap* in wild type and *Mst-/*- ES cells. *Actin* was analyzed as an internal control. The data are shown as the mean ± S.D (n=3). Statistically significant differences are indicated (*, P<0.05; **, P<0.01; ***, P<0.001). (B) Immunofluorescence staining of Yap and phosphorylated YapS127 in wild type ES cells and *Mst-/*- ES cells. Scale bar, 200 μm. (C) Quantitative RT-PCR to check mRNA level of *Ctgf* and *Cyr61* in wild type and *Mst-/*- ES cells. *Actin* was analyzed as an internal control. The data are shown as the mean ± S.D (n=3). Statistically significant differences are indicated (*, P<0.05; **, P<0.01; ***, P<0.001).
(TIF)

Figure S3 **Examination of *Mst-/-* ES cell pluripotency by embryo injection and teratoma formation.** (A) GFP labeled *Mst-/*- ES cells were integrated into ICM (arrow indicated in right panel) of blastocyst after aggregation with 8-cell stage embryos. Scale bar, 200 μm. (B) Teratoma formed by wild type ES cells 6 weeks after subcutaneous injection of wild type ES cells into nude mice (indicated by a white box). H&E staining showed tissue of three germ layers (ectoderm, mesoderm and endoderm). No teratomas were formed by subcutaneous injection of *Mst-/*- ES cells. Scale bar, 200 μm. (C) Quantitative RT-PCR to check mRNA level of *Mst1*, *Mst2* and *Yap* in ES cells and teratomas. *Actin* was analyzed as an internal control. The data are shown as the mean ± S.D (n=3). Statistically significant differences are indicated (*, P<0.05; **, P<0.01; ***, P<0.001). (D) Heatmap to show the expression of Foxo genes (*Foxo1*, *3a* and *4*), proapoptotic, antiapoptotic genes and tumor marker genes in day 8 wild type EBs and *Mst-/*- EBs.
(TIF)

Figure S4 **Pluripotency and lineage marker expression during *Mst-/-* EB formation.** (A) Immunoblotting and densitometric analysis to check the protein level of Pax6, Gata6 and T in day 4 and day 8 wild type EBs and Mst-/- EBs. Gapdh1 was analyzed as an internal control. The data are shown as the mean ± S.D (n=2). Statistically significant differences are indicated (*, P<0.05; **, P<0.01; ***, P<0.001). (B) Heatmap to show the expression of pluripotent genes and lineage genes (Ectoderm, Mesoderm and Endoderm) in day 4 and day 8 wild type EBs and *Mst-/*- EBs.
(TIF)

Figure S5 **The expression of ectoderm and endoderm lineage markers during *Mst-/-* EB formation.** (A) Phase contrast pictures of differentiated neural progenitor cells grown from wild type EBs and *Mst-/*- EBs at day 2 (top) and day 8 (middle) after attaching day 4 EBs to the plate (top) and the 2^nd^ passage culture of neural progenitor cells (bottom). Scale bar, 200 μm. (B) Immunofluorescence staining with antibody against Pax6 to examine the expression of Pax6 in wild type ES cells and *Mst-/*- ES cells cultured in neural differentiation medium for 8 days. Scale bar, 200 μm. (C) Heatmap to show the expression of cell markers of ectoderm and endoderm differentiated tissue cells in day 4 and day 8 wild type EBs and *Mst-/*- EBs. (D) Immunoblotting analysis to check hepatocyte markers (Albumin and AAT) in day 4 and day 8 wild type EBs and *Mst-/*- EBs. Gapdh1 was analyzed as an internal control.
(TIF)

Figure S6 **Comparison of proliferation difference between wild type ES cells and *Mst-/*- ES cells.** (A) Quantitative RT-PCR to check mRNA level of *Ccnd2* and *Ccnd3*, in wild type ES cells and *Mst-/*- ES cells. *Actin* was analyzed as an internal control. The data are shown as the mean ± S.D (n=3). Statistically significant differences are indicated (*, P<0.05; **, P<0.01; ***, P<0.001). (B) Flow diagram of BrdU labeled wild type ES cells and *Mst-/*- ES cells. The percentage of BrdU positive cell was marked in the diagram. (C) Percentage of BrdU positive cells in wild type ES cells and *Mst-/*- ES cells. Results represent the mean± S.D (n=2). Statistically significant differences are indicated (*, P<0.05; **, P<0.01; ***, P<0.001).
(TIF)

Figure S7 **The expression of mesoderm lineage markers during *Mst-/-* EB formation.** (A) Heatmap to show the expression of mesoderm differentiated tissue genes in day 4 and day 8 wild type EBs and *Mst-/*- EBs. (B) Quantitative RT-PCR to validate microarray results and check mRNA level of cardiomyocyte marker genes (*Tnnt2* and *Mef2c*), endothelial cell marker genes (*Pecam1* and *Cdh5*), Smooth muscle cell marker gene (*Acta2*), skeletal muscle cell marker gene (*MyoD1*) and hematopoietic stem cell marker genes (*Tal1* and *CD34*) in wild type and *Mst-/*- EB cells. *Actin* was analyzed as an internal control. The data are shown as the mean ± S.D (n=3). Statistically significant differences are indicated (*, P<0.05; **, P<0.01; ***, P<0.001).
(TIF)

Movie S1 **Wild type EBs grown in cardiac differentiation medium.**
(AVI)

Movie S2 ***Mst-/-* EBs grown in cardiac differentiation medium.**
(AVI)

Table S1 **Genotyping and quantitative RT-PCR primer list.**
(XLS)
