# Supplementary figures and images for: Functional Role of Mst1/Mst2 in Embryonic Stem Cell Differentiation
Source: PLoS One. 2013 Nov 5;8(11):e79867. doi: 10.1371/journal.pone.0079867 (PMC3818222; doi:10.1371/journal.pone.0079867)

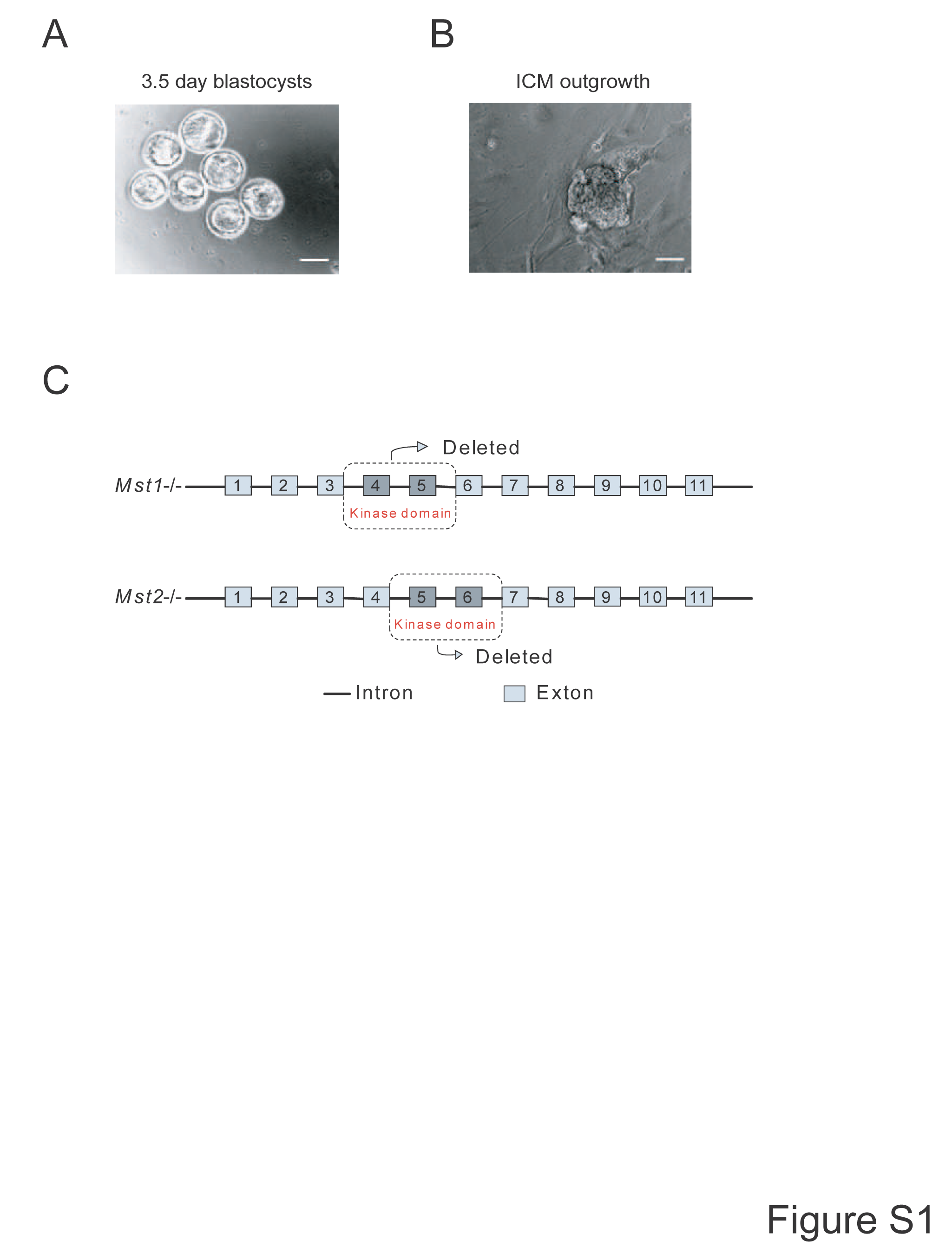

Supplement: Figure S1 — Mst-/- ES cell derivation. (A) 3.5 day blastocysts obtained by crossing Mst1+/-, Mst2-/- male and female mice. Scale bar, 200μm. (B) ICM outgrowth formed 5 days after a single blastocyst was seeded on MEF feeder in 2i+LIF ES medium. Scale bar, 200μm. (C) Schematics of targeted deletion loci (kinase domain) of Mst1 and Mst2. Boxes denote exons and lines denote intron. (TIF) [file pone.0079867.s001.tif]

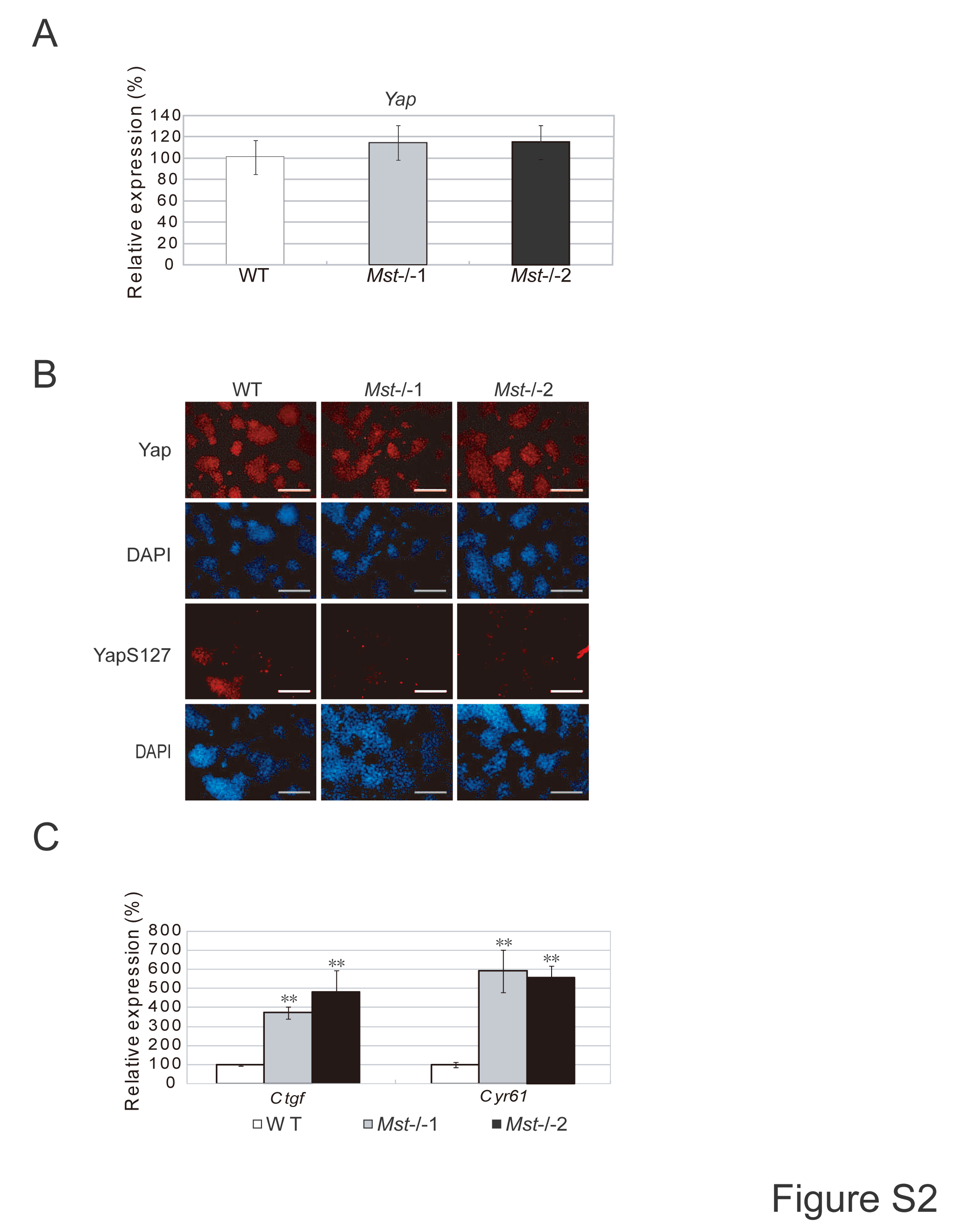

Supplement: Figure S2 — The expression of Yap and Yap targets in Mst-/- ES cells. (A) Quantitative RT-PCR to check mRNA level of Yap in wild type and Mst-/- ES cells. Actin was analyzed as an internal control. The data are shown as the mean ± S.D (n=3). Statistically significant differences are indicated (*, P<0.05; **, P<0.01; ***, P<0.001). (B) Immunofluorescence staining of Yap and phosphorylated YapS127 in wild type ES cells and Mst-/- ES cells. Scale bar, 200 μm. (C) Quantitative RT-PCR to check mRNA level of Ctgf and Cyr61 in wild type and Mst-/- ES cells. Actin was analyzed as an internal control. The data are shown as the mean ± S.D (n=3). Statistically significant differences are indicated (*, P<0.05; **, P<0.01; ***, P<0.001). (TIF) [file pone.0079867.s002.tif]

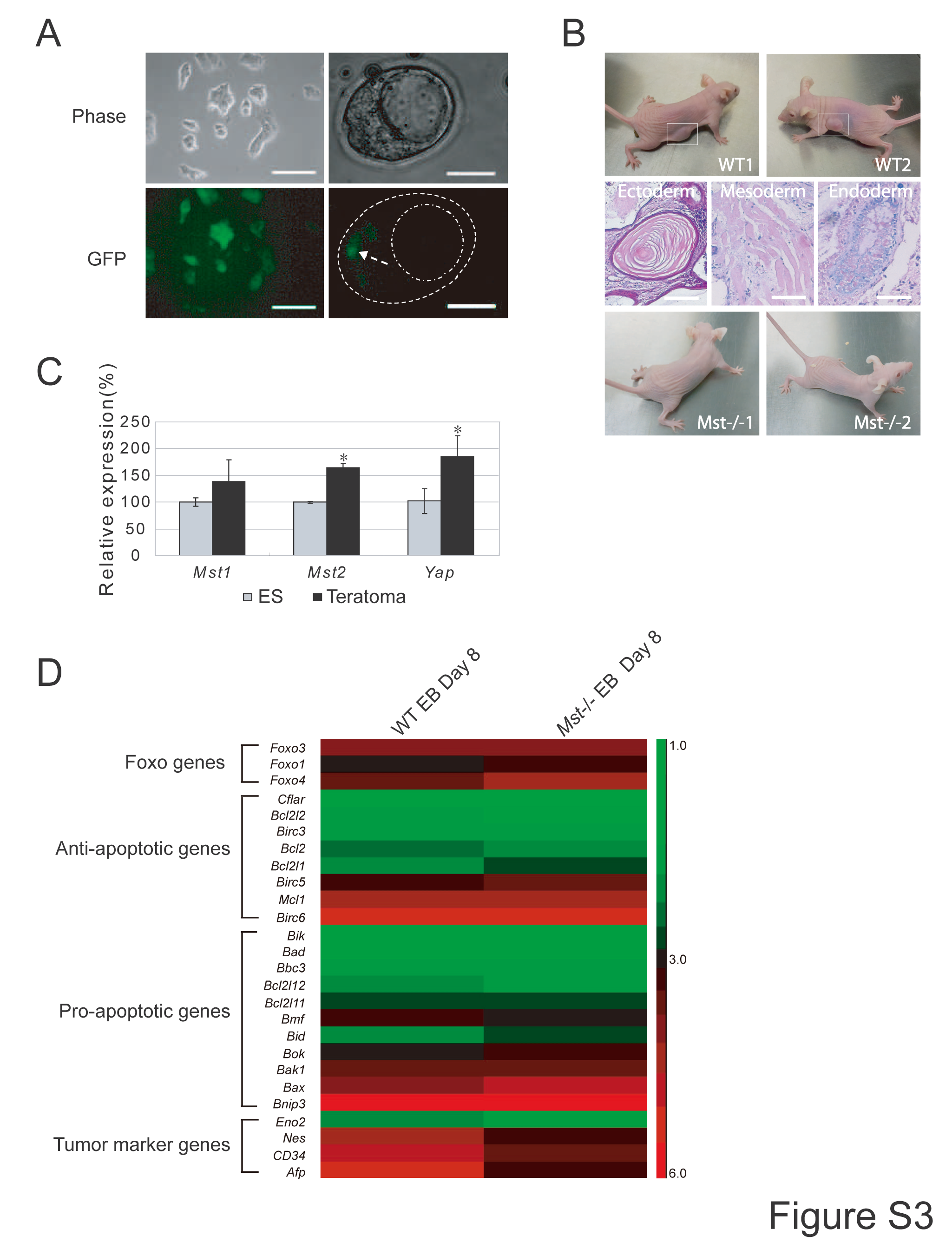

Supplement: Figure S3 — Examination of Mst-/- ES cell pluripotency by embryo injection and teratoma formation. (A) GFP labeled Mst-/- ES cells were integrated into ICM (arrow indicated in right panel) of blastocyst after aggregation with 8-cell stage embryos. Scale bar, 200 μm. (B) Teratoma formed by wild type ES cells 6 weeks after subcutaneous injection of wild type ES cells into nude mice (indicated by a white box). H&E staining showed tissue of three germ layers (ectoderm, mesoderm and endoderm). No teratomas were formed by subcutaneous injection of Mst-/- ES cells. Scale bar, 200 μm. (C) Quantitative RT-PCR to check mRNA level of Mst1, Mst2 and Yap in ES cells and teratomas. Actin was analyzed as an internal control. The data are shown as the mean ± S.D (n=3). Statistically significant differences are indicated (*, P<0.05; **, P<0.01; ***, P<0.001). (D) Heatmap to show the expression of Foxo genes (Foxo1, 3a and 4), proapoptotic, antiapoptotic genes and tumor marker genes in day 8 wild type EBs and Mst-/- EBs. (TIF) [file pone.0079867.s003.tif]

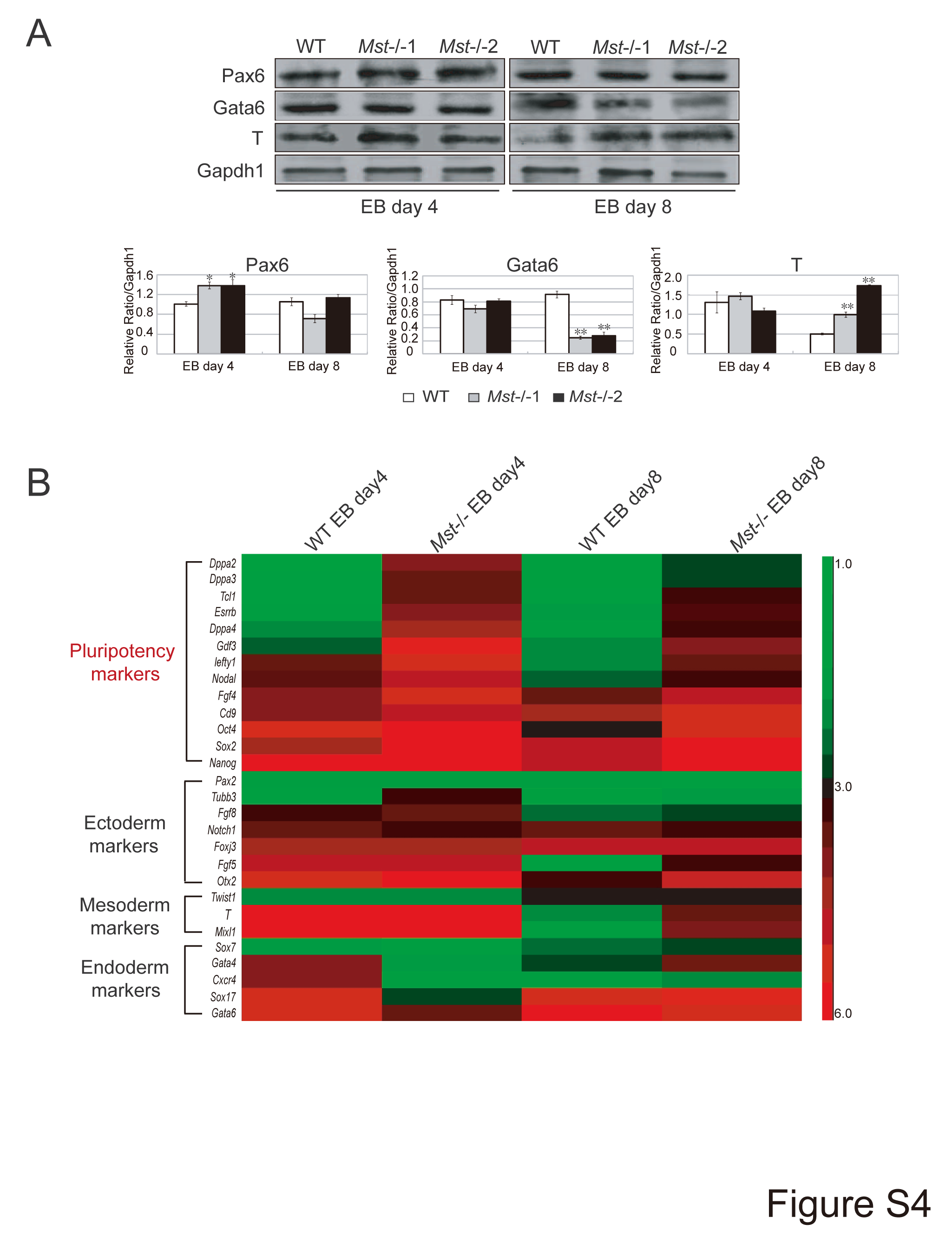

Supplement: Figure S4 — Pluripotency and lineage marker expression during Mst-/- EB formation. (A) Immunoblotting and densitometric analysis to check the protein level of Pax6, Gata6 and T in day 4 and day 8 wild type EBs and Mst-/- EBs. Gapdh1 was analyzed as an internal control. The data are shown as the mean ± S.D (n=2). Statistically significant differences are indicated (*, P<0.05; **, P<0.01; ***, P<0.001). (B) Heatmap to show the expression of pluripotent genes and lineage genes (Ectoderm, Mesoderm and Endoderm) in day 4 and day 8 wild type EBs and Mst-/- EBs. (TIF) [file pone.0079867.s004.tif]

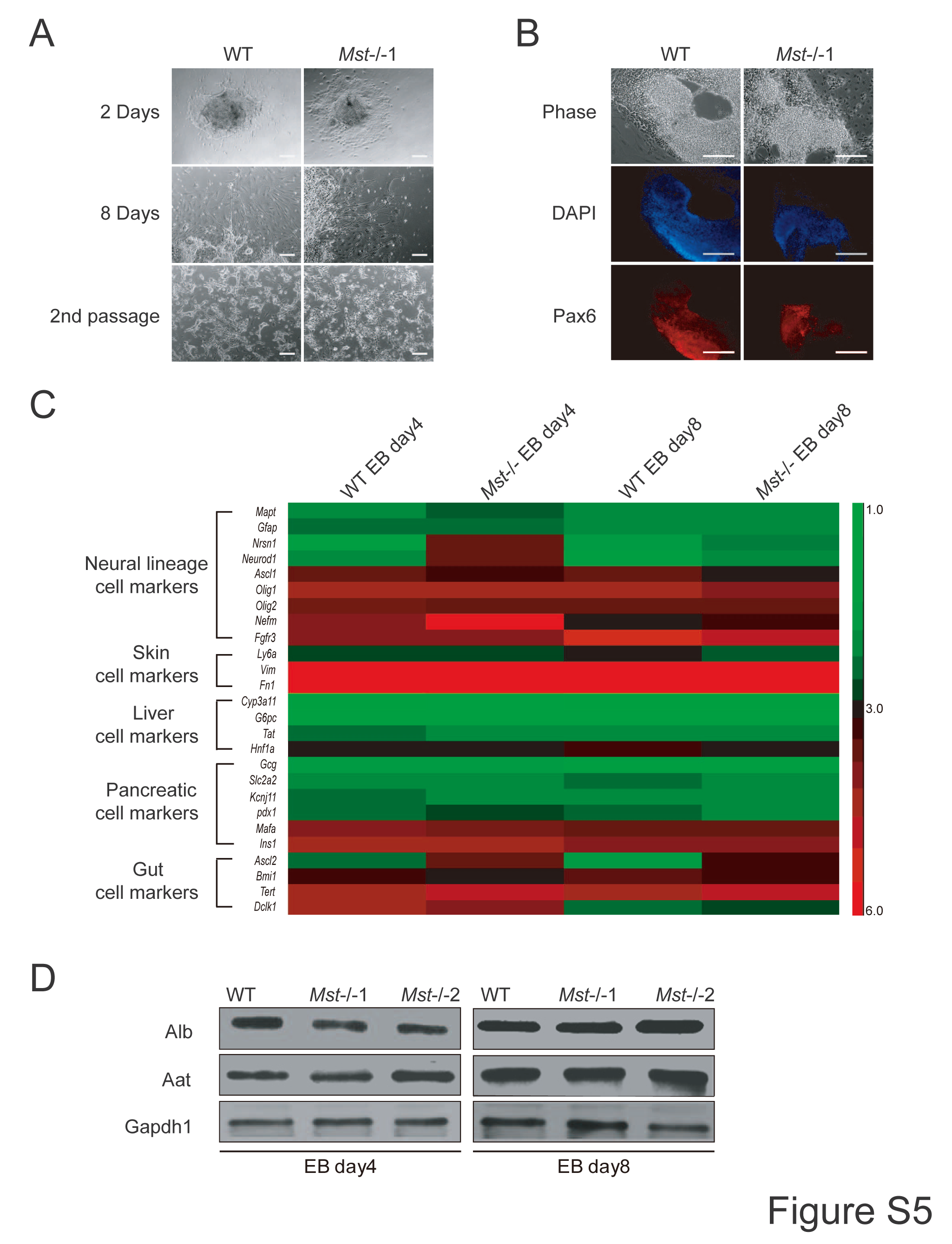

Supplement: Figure S5 — The expression of ectoderm and endoderm lineage markers during Mst-/- EB formation. (A) Phase contrast pictures of differentiated neural progenitor cells grown from wild type EBs and Mst-/- EBs at day 2 (top) and day 8 (middle) after attaching day 4 EBs to the plate (top) and the 2nd passage culture of neural progenitor cells (bottom). Scale bar, 200 μm. (B) Immunofluorescence staining with antibody against Pax6 to examine the expression of Pax6 in wild type ES cells and Mst-/- ES cells cultured in neural differentiation medium for 8 days. Scale bar, 200 μm. (C) Heatmap to show the expression of cell markers of ectoderm and endoderm differentiated tissue cells in day 4 and day 8 wild type EBs and Mst-/- EBs. (D) Immunoblotting analysis to check hepatocyte markers (Albumin and AAT) in day 4 and day 8 wild type EBs and Mst-/- EBs. Gapdh1 was analyzed as an internal control. (TIF) [file pone.0079867.s005.tif]

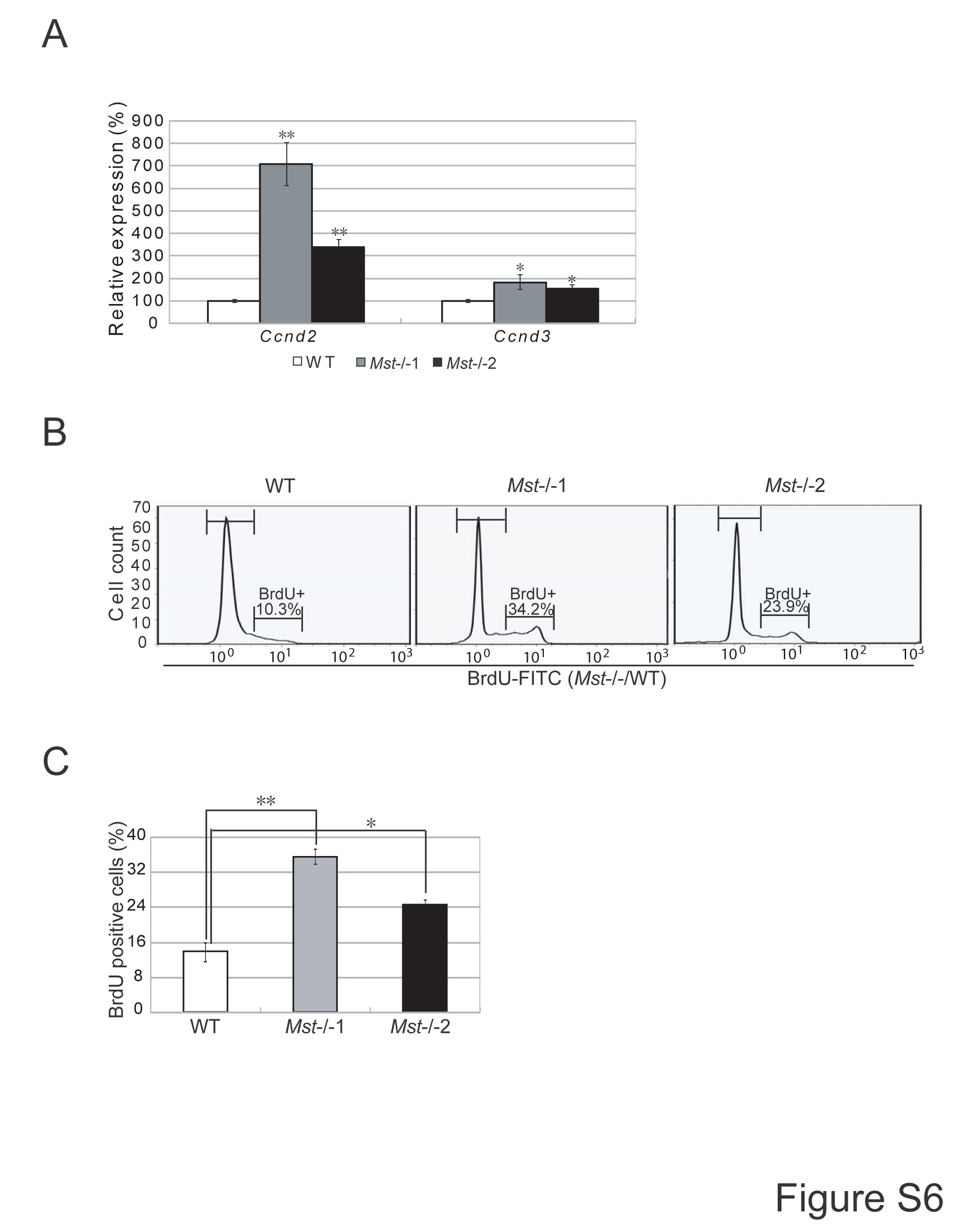

Supplement: Figure S6 — Comparison of proliferation difference between wild type ES cells and Mst-/- ES cells. (A) Quantitative RT-PCR to check mRNA level of Ccnd2 and Ccnd3, in wild type ES cells and Mst-/- ES cells. Actin was analyzed as an internal control. The data are shown as the mean ± S.D (n=3). Statistically significant differences are indicated (*, P<0.05; **, P<0.01; ***, P<0.001). (B) Flow diagram of BrdU labeled wild type ES cells and Mst-/- ES cells. The percentage of BrdU positive cell was marked in the diagram. (C) Percentage of BrdU positive cells in wild type ES cells and Mst-/- ES cells. Results represent the mean± S.D (n=2). Statistically significant differences are indicated (*, P<0.05; **, P<0.01; ***, P<0.001). (TIF) [file pone.0079867.s006.tif]

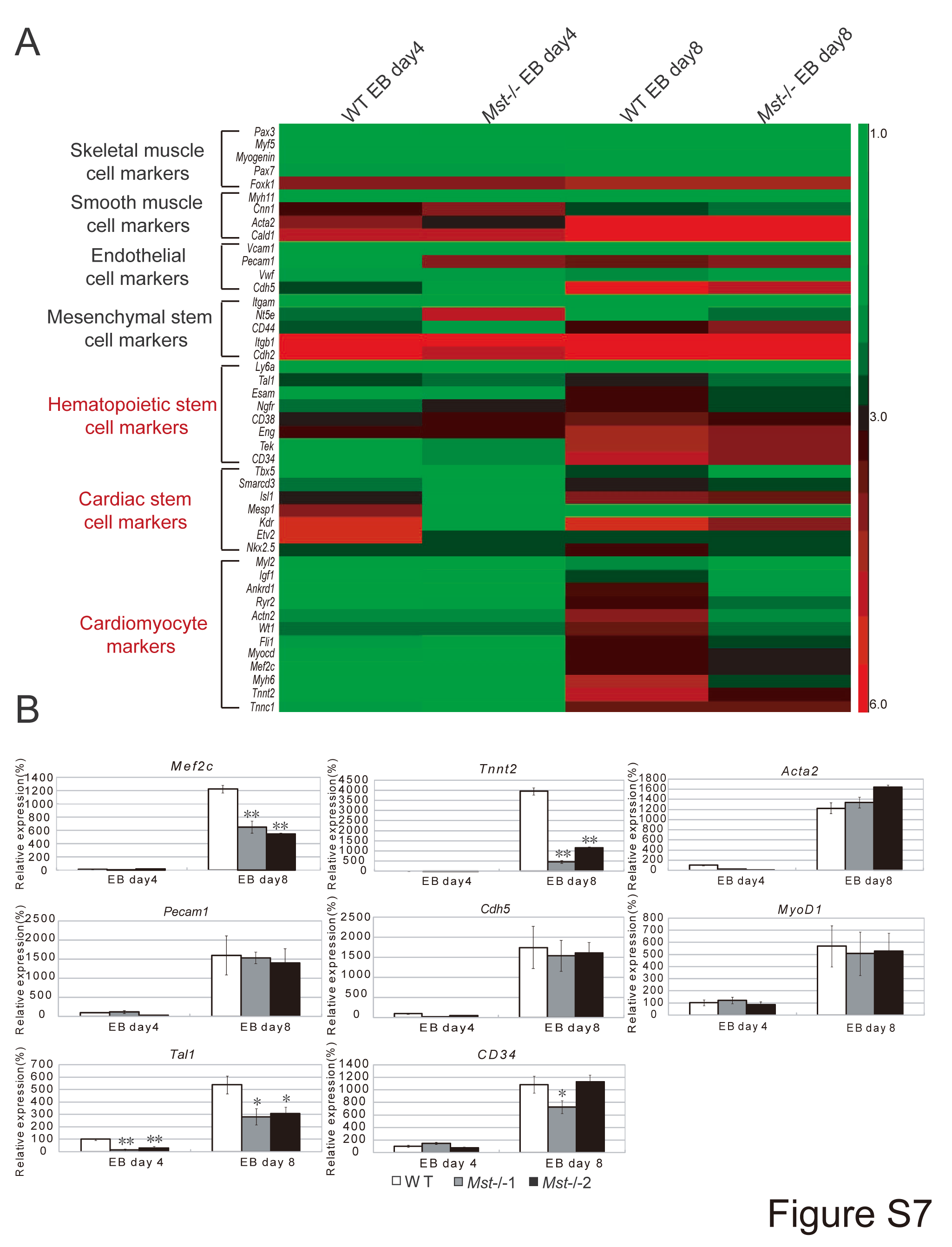

Supplement: Figure S7 — The expression of mesoderm lineage markers during Mst-/- EB formation. (A) Heatmap to show the expression of mesoderm differentiated tissue genes in day 4 and day 8 wild type EBs and Mst-/- EBs. (B) Quantitative RT-PCR to validate microarray results and check mRNA level of cardiomyocyte marker genes (Tnnt2 and Mef2c), endothelial cell marker genes (Pecam1 and Cdh5), Smooth muscle cell marker gene (Acta2), skeletal muscle cell marker gene (MyoD1) and hematopoietic stem cell marker genes (Tal1 and CD34) in wild type and Mst-/- EB cells. Actin was analyzed as an internal control. The data are shown as the mean ± S.D (n=3). Statistically significant differences are indicated (*, P<0.05; **, P<0.01; ***, P<0.001). (TIF) [file pone.0079867.s007.tif]
